# Functional panchromatic BODIPY dyes with near-infrared absorption: design, synthesis, characterization and use in dye-sensitized solar cells

**DOI:** 10.3762/bjoc.15.169

**Published:** 2019-07-24

**Authors:** Quentin Huaulmé, Cyril Aumaitre, Outi Vilhelmiina Kontkanen, David Beljonne, Alexandra Sutter, Gilles Ulrich, Renaud Demadrille, Nicolas Leclerc

**Affiliations:** 1 CEA, Univ. Grenoble Alpes, CNRS, IRIG, SyMMES, F-38000 Grenoble, France; 2 Chimie des Matériaux Nouveaux & Centre d'Innovation et de Recherche en Matériaux Polymères Université de Mons - UMONS / Materia Nova Place du Parc, 20, B-7000 Mons, Belgium; 3 ICPEES – UMR7515, CNRS-Université de Strasbourg, 25 rue Becquerel, 67087 Strasbourg CEDEX 02, France

**Keywords:** boron-dipyrromethene, BODIPY, dye-sensitized solar cells, near-infrared absorbers, organic dyes

## Abstract

We report two novel functional dyes based on a boron-dipyrromethene (BODIPY) core displaying a panchromatic absorption with an extension to the near-infrared (NIR) range. An innovative synthetic approach for preparing the 2,3,5,6-tetramethyl-BODIPY unit is disclosed, and a versatile way to further functionalize this unit has been developed. The optoelectronic properties of the two dyes were computed by density functional theory modelling (DFT) and characterized through UV–vis spectroscopy and cyclic voltammetry (CV) measurements. Finally, we report preliminary results obtained using these functional dyes as photosensitizers in dye-sensitized solar cells (DSSCs).

## Introduction

The past two decades have witnessed tremendous efforts to develop alternative photovoltaic (PV) technologies. Among them, dye-sensitized solar cells (DSSCs) display numerous advantages compared to its fully organic counterpart, i.e., bulk heterojunction solar cells, or other hybrid PV technologies such as perovskite solar cells. DSSCs can display satisfactory power conversion efficiencies (PCE) in the range of 10 to 14% [1–3] but also a long-term stability when specific electrolytes based on ionic liquids are employed [4–5]. Besides, this technology enables the fabrication of solar panels that can be prepared semi-transparent, colorful, and out of non-toxic constituents [6]. Historically, Ru(II)–polypyridyl complexes were the most used dyes as photosensitizers in DSSCs (N719 or N749 Black Dye) [7] but these molecules reveal low absorption coefficients over the visible range. Furthermore, they contain a rare and a relatively high-cost element (Ru) and their plausible toxicity restrains their development at the industrial level. For these reasons, in the last decade, metal-free organic dyes based on donor–(π-spacer)–acceptor structure have hence been extensively investigated and screened as sensitizers in DSSCs. Among the hundreds of dyes developed so far for this application, only few can show panchromatic absorption [8–9]. To improve the photogeneration of current, and hence the efficiency of DSSCs, the development of new dye molecules better matching the solar emission spectrum or exhibiting absorption in the near-infrared (NIR) range could be a fruitful strategy.

Several families of molecules displaying an infrared absorption have been investigated in the last decade, such as phthalocyanines [10–11], organic push–pull compounds [12], and boron-dipyrromethene [13] (BODIPY^®^). BODIPY dyes are one of the most extensively studied class of fluorophores due to their unique properties, including high absorption coefficients in the visible and NIR ranges, high fluorescence quantum yields, and high stability in various media. More importantly, they display a very versatile chemistry, allowing the fine tuning of all their physical and optical properties [14]. They hence have found applications in various fields, such as lasers dyes [15], (bio)-labeling [16–17], photodynamic therapy [18], or even bulk heterojunction solar cells [19–20] or DSSCs [21–22].

In this articl we report an innovative synthetic approach for synthesizing 2,3,5,6-tetramethyl-BODIPY compounds and a way to further functionalize such cores has been developed. The optoelectronic properties of the functional molecules were investigated using UV–vis spectroscopy, and we show that they can absorb light up to 900 nm in solution and 1000 nm in solid state, after grafting on anatase-TiO_2_ mesoporous films. The cyclic voltammetry (CV) measurements indicate that the compounds have HOMO and LUMO energy levels suitable for an application in DSSCs, in rather good agreement with the values obtained from DFT calculations. Finally, we report preliminary results employing these molecules as photosensitizers in dye solar cells with iodine-based liquid electrolytes. We show that the limited performances of these new BODIPY derivatives arise from their deep LUMO energy level. In terms of energy, the latter lies close to the conduction band of the electron-transporting oxide, limiting therefore the driving force of electronic injection, and hence the overall efficiency of the resulting solar cell.

## Results and Discussion

### 1. Design and DFT calculations

**Figure 1:**
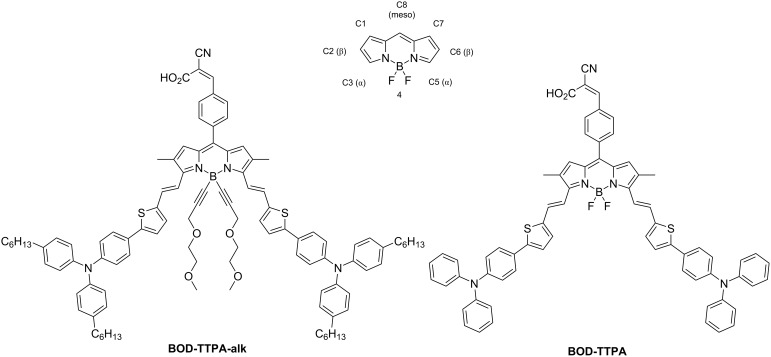
Molecular structures of the two target compounds **BOD-TTPA-alk** and **BOD-TTPA,** and the chemical structure of a BODIPY core displaying its positions numbering.

In many opto-electronic devices the light-absorption properties of the semiconductors are a critical parameter. This is particularly the case when solar energy conversion applications are targeted. For instance, in order to maximize the photocurrent density in a DSSC device, the sensitizer has to display high molar absorption coefficients, ideally along the entire visible range. Additionally, it must display appropriate positioning of frontier molecular orbitals (highest occupied molecular orbital/lowest unoccupied molecular orbital, HOMO/LUMO) energy levels with respect to the conduction band (CB) of the metal oxide and the redox potential of the electrolyte. Abundant literature on BODIPY derivatives allowed us to identify a chemical approach to fulfill those criteria, i.e., the introduction of an electron rich unit at the 3rd and 5th positions of the BODIPY core via a vinyl spacer [14,21,23–24]. Introduction of such units on those positions is known to lead to a larger bathochromic shift of the S_0_→S_1_ absorption band than the same substitution on its 2nd and 6th positions [25]. A thiophene-triphenylamine unit was selected among the reported electron-donating units, due to its reversible redox properties and high electron-donating strength (see Figure 1) [26]. In order to promote the delocalization from the electron-donating unit to the electron-withdrawing and anchoring group located on its 8 position, a 2,3,5,6-tetramethyl-BODIPY derivative has been designed (see Figure 1). Indeed, most of the BODIPY based materials, used in organic semiconducting applications, exhibit methyl groups in the 1,7-positions. However, such groups in these positions hinder sterically the position 8 of BODIPY cores and therefore lead to a severe twist of the aromatic unit grafted on. Removing these methyl groups is an effective way to reduce the dihedral angle between the meso substituent and the BODIPY core by lowering the steric hindrance between the two latter. It has been previously reported that a better molecular planarity usually originates a higher charge injection efficiency [27]. Furthermore, it has been reported that introduction of hydrophobic alkyl chains on a sensitizer is a way to improve the open-circuit voltage (V_oc_), by reducing the electronic recombination rate at the electrolyte/semi-conducting oxide interface [28]. To probe the validity of this concept for a BODIPY derivative, we decided to synthetize the two molecular structures disclosed in Figure 1, namely **BOD-TTPA-alk** and **BOD-TTPA**.

**Figure 2:**
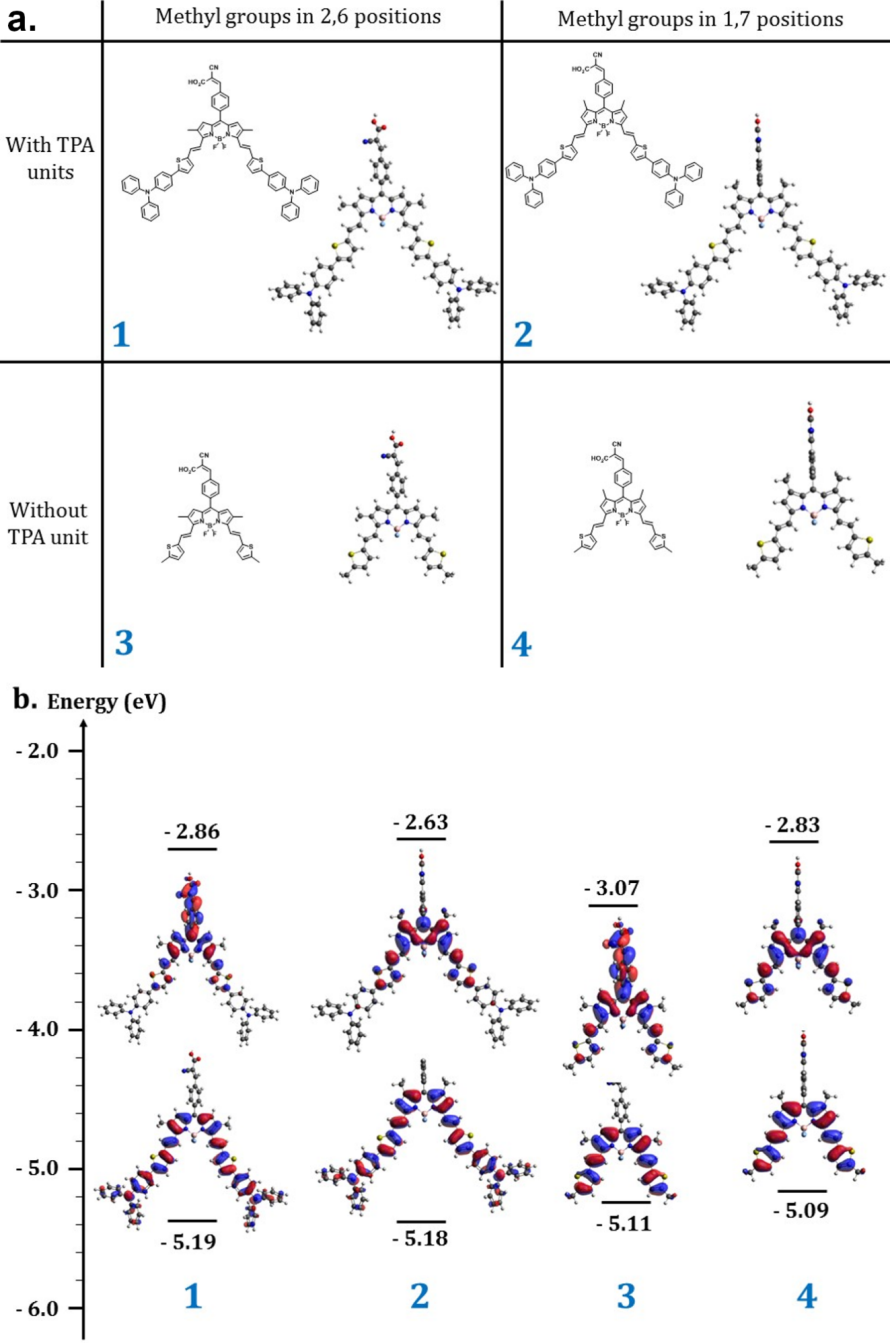
a) Geometrical optimization of four representative BODIPY-based materials for DSSCs application. b) Frontiers molecular orbitals repartition and their predicted energy values for the selected four representative BODIPY-based dyes.

To support our molecular design, and especially to have better insight on the electronic structure and optical properties of the dyes, we performed (time-dependent) density functional theory (TD-DFT) calculations on the four representative molecules displayed in Figure 2: Dyes (**1** and **3**) vs (**2** and **4**) differ by the position (2,6 or 1,7) of the methyl groups on the BODIPY core, while dyes (**1** and **2**) vs (**3** and **4**) differ by the presence (or not) of a triphenylamine donor group on the thiophene ring. As we are dealing with charge-transfer electronic excitations, we have adopted a tuned range-separated CAM-B3LYP functional and the polarizable continuum model (PCM) to account for (implicit) solvent effects (in THF).

The one-electron energy diagram reported in Figure 2 shows that: (i) Grafting the triphenylamine donor moieties on the thiophene-vinylene bridge only slightly (by ca. 0.1 eV) lowers the ionization potential of the dyes, compare (**1**,**2**) to (**3**,**4**); this is explained by the fact that the HOMO molecular orbital mostly spreads on the BODIPY-vinylthiophene core of the molecules, with only slight contributions from the TPA units (ii) The position of the methyl groups has by far a larger impact on the electron affinity of the molecules, which is substantially increased when going from **2** to **1**, and from **4** to **3**. This is clearly not a direct electronic effect but rather results from the close to orthogonal orientation of the anchoring groups induced by steric effects in **2** and **4**. As a result, while the LUMO orbital largely extends through the BODIPY unit towards the cyanoacrylic acid anchors in the case of **1** and **3**, it is completely confined to the BODIPY-vinylthiophene core in **2** and **4**, explaining the deeper unoccupied levels in the former molecules.

**Figure 3:**
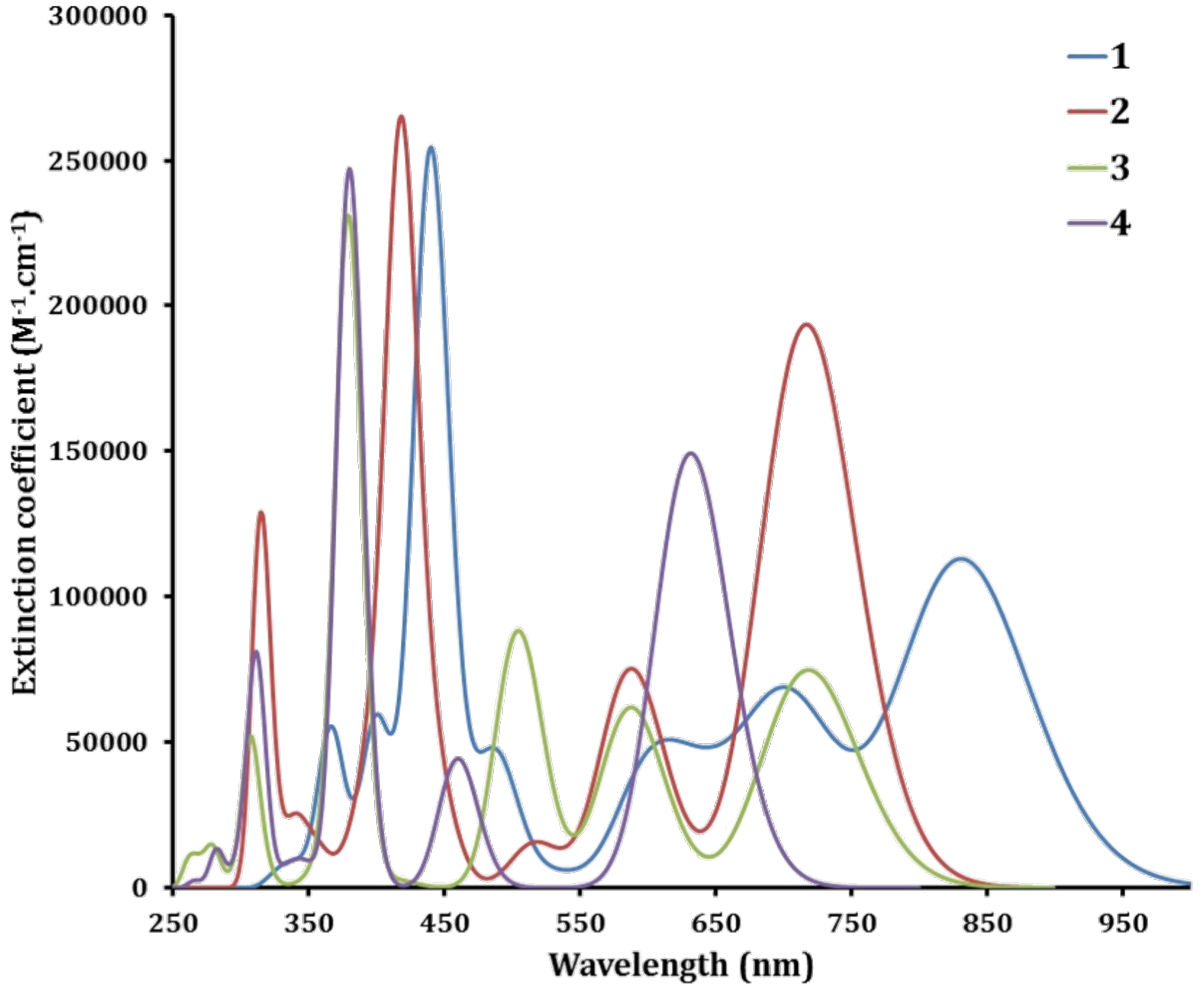
Predicted absorption spectra of the four dyes.

Thus, our calculations suggest that, in case of an allowed HOMO–LUMO transition, **1** and **3** should show a bathochromically shifted optical absorption spectrum, thereby hopefully allowing for a more efficient sunlight absorption, yet also a reduced energy driving force for charge separation at the TiO_2_ surface. To check this hypothesis, we performed TD-DFT simulations of the optical absorption spectra of the 4 representative dyes. The results reported in Figure 3 are fully consistent with the predictions from the one-electron picture. There is a substantial red shift of the lowest optical absorption band, from ≈750 nm in **2** to ≈850 nm in **1** by moving the methyl groups from (1,7) to (2,6) positions, in line with the increased electronic delocalization over the anchoring groups. An additional virtue of the placement of the methyl groups in positions (2,6) is to extend the absorption range of the dyes across the whole visible range. Very similar effects are observed for the TPA-free molecules, except for an overall blue shift of the main absorption bands resulting from the reduced size of the π-electronic system.

### 2. Synthesis

**Figure 4:**
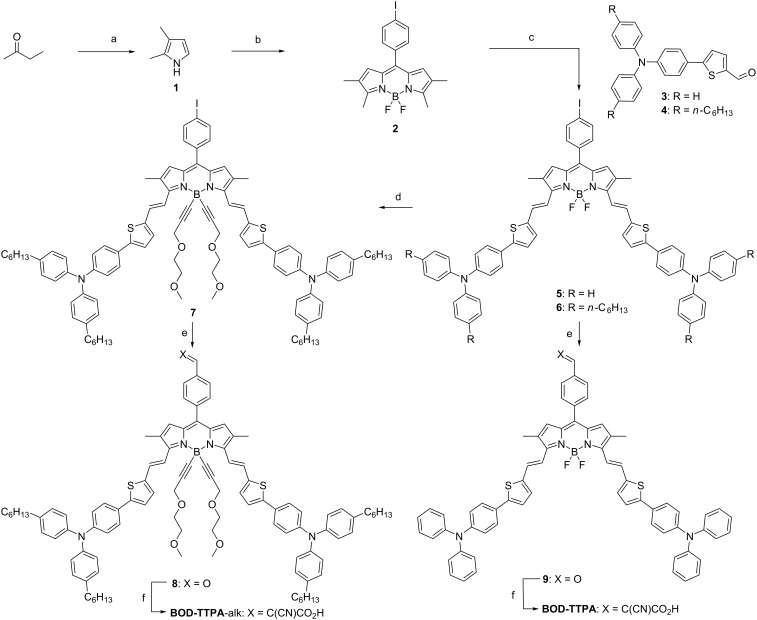
Synthetic scheme of the selected materials. a) hydroxylamine hydrochloride, NaHCO_3_, DMSO, 60 °C then acetylene, KOH, DMSO, 110 °C, 24% over the two steps; b) 4-iodobenzoyl chloride, DCM, rt then DDQ, DCM, rt then NEt_3_, BF_3_·OEt_2_, 0 °C to rt, 35%; c) piperidine, cat. PTSA, toluene, 130 °C, **5**: 35%, **6**: 30%; d) (3-(2-methoxyethoxy)prop-1-yn-1-yl)magnesium bromide, THF, 60 °C, 85%; e) carbon monoxide, sodium formiate, [Pd(PPh_3_)_2_Cl_2_], anhydrous DMF, 100 °C, **8**: 19%, **9**: 35%; f) cyanoacetic acid, piperidine, MeCN, CHCl_3_, 80 °C, **BOD-TTPA**: 34%, **BOD-TTPA-alk**: 26%.

The synthesis of the target materials is schemed in Figure 4. To introduce the selected substitution pattern, 2,3-dimethylpyrrole **1** is first synthesized through a Trofimov reaction in a one-pot two-step reaction [29]. The condensation of the aforementioned pyrrole on *para*-iodobenzoyl chloride affords the corresponding dipyrromethenium chloride, which was then converted into its BODIPY analogue **2** through complexation by BF_3_·OEt_2_ in basic media. Regioselective introduction of distyryl substituents is achieved via Knoevenagel-type condensation in the presence of piperidine using aldehyde derivatives **3** and **4** whose synthesis are described in Supporting Information File 1. This condensation reaction affords compounds **5** and **6** with a slight variation of their pendant alkyl chains. The stereoisomerism of the resulting distyryl compound (*trans*) has been unambiguously attributed by ^1^H NMR (see Figures **S9** and **S11** in Supporting Information File 1), whose spectra feature characteristic constant couplings of 16.2 and 16.1 Hz. It is worth mentioning that the fluorine substitution was performed on the boron center after introduction of the styryl residues, introduction prior to the Knoevenagel reaction is known to impede it [30]. The introduction of the aldehyde moiety is carried out through catalytic carbo-palladation reaction using molecular carbon monoxide in the presence of methyl formiate as hydrogen source in modest yield (35% and 19% for **8** and **10**, respectively) due to the formation of a relatively large amount of dehalogenation side-product. Finally, a Knoevenagel condensation in the presence of cyanoacetic acid and piperidine is performed to lead to the targeted compounds **BOD-TTPA-alk** and **BOD-TTPA**.

### 3. Optical properties

**Figure 5:**
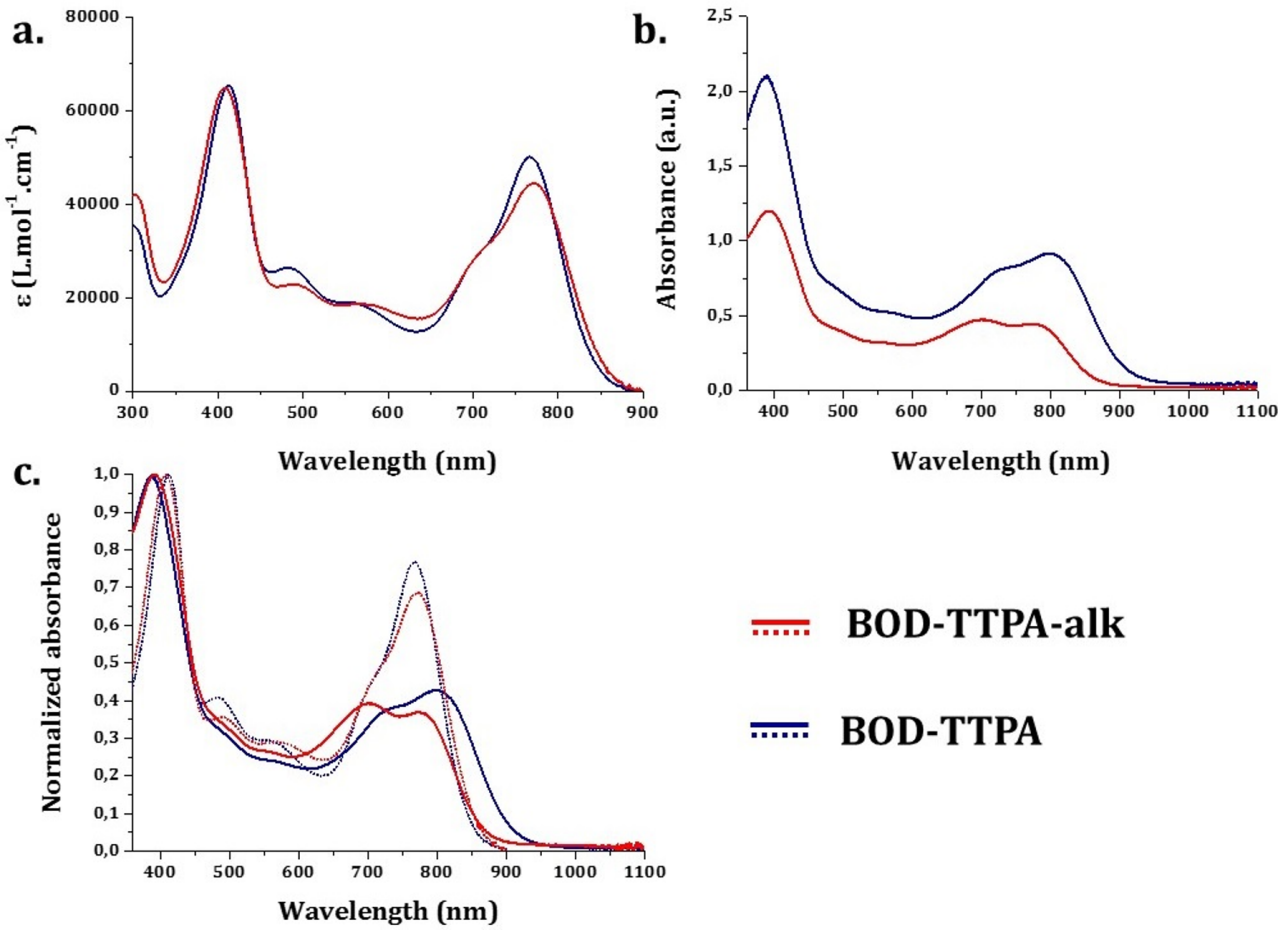
a) Absorption spectra of compounds **BOD-TTPA-alk** and **BOD-TTPA** (THF, ≈10^−6^ M, 25 °C). b) Absorbance spectra of 2 µm thick mesoporous TiO_2_ sensitized in a solution of dye/CDCA (1:10, molar ratio). c) Normalized absorbance spectra of **BOD-TTPA-alk** (red traces) and **BOD-TTPA** (blue traces), both in diluted solution (aerated THF, ≈10^−6^ M, 25 °C – dashed lines) and once adsorbed on 2 µm thick mesoporous TiO_2_ (solid lines).

The optical properties of compounds **BOD-TTPA-alk** and **BOD-TTPA** were first evaluated in diluted (≈10^−6^ M in THF) solution (see Figure 5). They both display two intense absorption bands. At lower energy, the absorption band displays a rather large and high extinction coefficient (5·10^−4^ M^−1^cm^−1^) band attributed to the S_0_→S_1_ transition of the BODIPY core, and still displays its characteristic shoulder at higher energy [14]. Another intense absorption band is located around 400 nm and is attributed to the π→π* transition of the triphenylamine thiophene residues. It is worth noting that almost no difference is observed between those two materials, which is consistent with their identical electronic structure. As often with such distyryl-BODIPY derivatives [19,25], no clear cut-off of absorbance is observed in the whole part of the visible range, leading to a panchromatic absorption. The absorption is not limited to the visible domain, indeed the compound **BOD-TTPA-alk** and **BOD-TTPA** exhibit a λ_max_ in solution located at 771 nm and 768 nm, respectively, with an absorption edge close to 900 nm. The absorption in the NIR region is a peculiar property that is of course a considerable advantage for solar cell applications.

With the aim of investigating the behavior of those dyes once adsorbed on mesoporous TiO_2_, sensitization has been performed on 2 µm thick TiO_2_ mesoporous layer (see Figure 5b and c). To prevent dye aggregation on the surface of the semi-conducting oxide, and to mimic the real conditions of use in solar cells, chenodeoxycholic acid (CDCA) was employed as co-sensitizer and hence added to the sensitization solution [31]. For the dyeing solutions the concentration of the dye was 1 mM and the concentration of CDCA was 10 mM in a chloroform/ethanol mixture (1:1 in volume). The absorbance spectra of the resulting sensitized TiO_2_ layers are recorded and presented in Figure 5b.

For both dyes, grafting them on the surface clearly impacts the absorption bands attributed to the BODIPY part of the systems. The absorption coefficient of the lower energy absorption band, attributed to the S_0_→S_1_ transition, is reduced and a higher energy band around 700 nm increases in intensity. This last one is more likely attributed to a conformationally restricted form of the dye once adsorbed which shows an absorption value closed to mono-styryl species, thus reinforcing the panchromatic absorption of this dye. As long as **BOD-TTPA-alk** is concerned, no significant shift of the lower energy band is observed (see Table 1 and Figure 5a). This suggests no self-aggregation of the alkylated dye occurs on the oxide surface, which can be explained by the presence of the co-adsorbent CDCA, and of the multiple alkyl chain substitution of the TPA units. On the other hand, a bathochromic shift of the absorption profile of **BOD-TTPA** is observed from diluted solution to the anchored dye (see Figure 5a). Despite the same amount of CDCA in the dyeing solution (10 molar equivalents), this result suggests a higher tendency for π stacking interactions. Once anchored, **BOD-TTPA** exhibits consequently a broader absorption profile than its alkylated counterpart **BOD-TTPA-alk**.

**Table 1:** Selected optical properties of compounds **BOD-TTPA**-**alk** and **BOD-TTPA**.

Compounds	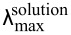 (nm)	ε (L·mol^−1^·cm^−1^) at 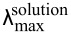	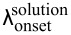 (nm)	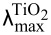 (nm)	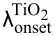 (cm)

**BOD-TTPA-alk**	409, 771	6.49·10^4^, 4.46·10^4^	841	393, 700, 771	867
**BOD-TTPA**	411, 768	6.52·10^4^, 5.00·10^4^	856	389, 801	907

Furthermore, the measured intensity of absorbance of the sensitized TiO_2_ layer is much higher for **BOD-TPA** than **BOD-TTPA-alk** in spite of the fact that the dyeing was performed with solutions containing exactly the same concentration of the dyes. The thickness of the aforementioned TiO_2_ layer being identical and the two dyes **BOD-TTPA-alk** and **BOD-TTPA** displaying very similar absorption coefficient (see Table 1), sensitization of mesoporous TiO_2_ will be more effective for **BOD-TTPA** than **BOD-TTPA-alk**. This lower absorption originating from a lower grafting level may arise from the overall size of the molecule **BOD-TTPA-alk** that displays 4 alkyl chains and 2 ethylene glycol chains. The bigger size of **BOD-TTPA-alk** can eventually prevents its diffusion through all the pores of the TiO_2_ layer or decreases the density of grafted molecules due to steric hindrance.

### 4. Electrochemical characterization

Cyclic voltammetry of both BODIPY dyes was carried out in deoxygenated DCM solutions containing tetrabutylammonium hexafluorophosphate as salt (see Figure S24 and S25 in Supporting Information File 1), to investigate their oxidation and reduction processes as well as to determine the energy levels of their highest occupied molecular orbital (HOMO) and lower unoccupied molecular orbital (LUMO). All the redox potentials were calibrated with respect to Ferrocene (Fc), assuming that *E*(Fc/Fc^+^) = 0.40 V with respect to SCE (see experimental details). The low solubility of the BOD-TTPA derivative makes this material much more difficult to characterize than its alkylated counterpart. However, both dyes exhibit similar first oxidation potentials about 0.6 V and 0.55 V for **BOD-TTPA** and **BOD-TTPA-alk**, respectively. Both processes are reversible. It worth noting that the **BOD-TTPA-alk** exhibits also a second reversible oxidation process at higher voltage of 0.8 V. Finally, for both dyes, a quasi-reversible reduction process could be observed at −0.6 V and −0.8 V for **BOD-TTPA** and **BOD-TTPA-alk**, respectively.

The HOMO and LUMO levels were determined by using the following equations (HOMO = *E*_ox_ + 4.4 eV) and (LUMO = *E*_red_ + 4.4 eV) where the onset potentials are in volts (vs SCE) and HOMO and LUMO are in electron volts [32]. We thus calculated HOMO and LUMO levels of −5.0 eV and −3.8 eV for **BOD-TTPA** against −4.95 eV and −3.6 eV for **BOD-TTPA-alk**. The small change of the HOMO energy levels is in line with the performed calculations (see section 1), where the HOMO molecular orbital is shown to be mostly spread on the BODIPY-vinylthiophene moieties of the dyes, with only slight contributions from the TPA units. The significant decrease in electronic affinity observed in **BOD-TTPA-alk** is likely due to the boron center alkylation. Similar effects have been already shown after fluorine substitution of BODIPY [33].

### 5. DSSCs fabrication and device performances

In order to investigate the photovoltaic performances of the two functional dyes **BOD-TTPA-alk** and **BOD-TTPA**, a set of DSSCs were fabricated following a procedure reported previously [4]. The *J*(V) characteristics of the devices were recorded in dark and upon irradiation with a mask. For the measurements a solar simulator with AM 1.5G filter was used after calibration with a Silicon cell at 1000 W m^−2^. For a direct comparison, we fabricated solar cells with the same photoelectrode composition consisting of a double layer TiO_2_ (a 12 µm-thick transparent layer and a 4 µm-thick scattering layer) purchased from Solaronix. This thickness was selected on the basis of a previous study showing that thicker electrodes (typically above 12 µm) give rise to highest photocurrent density.

When dyes with low-lying LUMO energy levels are employed as sensitizers together with TiO_2_ electrodes, the choice of the electrolyte is crucial. From CV experiments (see previous section) we found that the LUMO levels of the dyes are at −3.9 and −3.6 eV. In other words, they are located roughly 0.1–0.4 eV above the energy level of the conducting band of the oxide (which is around −4 eV). This alignment of the energy levels could be damaging to the electron injection process. Indeed, it is known that a minimum driving force of 0.15 eV (neglecting entropy changes during the light absorption) is required to efficiently inject photo excited electrons from the LUMO of the dyes in the CB of the oxide [34].

Usually iodine-based liquid electrolytes are comprising additives such as *tert*-butylpyridine (^t^BP) which is known to shift the CB band of the oxide positively by creating a dipole effect at the surface of TiO_2_. By suppressing this dipole effect, the CB of TiO_2_ could be relocated deeper and this could facilitate the photo-injection process. Consequently, for preliminary investigations we prepared an electrolyte with a formulation close to the one of HI-30 commercialized by Solaronix. HI-30 electrolyte is known to be compatible with a large variety of organic dyes. We therefore designed a modified electrolyte inspired from HI-30 with the following composition: 0.5 M of butylmethylimidazolium iodide (BMII), 0.03 M of I_2_ 0.5 M of LiI, 0.1 M of guanidinium thiocyanate in a mixture of acetonitrile and 3-methoxyproprionitrile (85:15). Compared to classical electrolytes, in this formulation we removed ^t^BP, hoping that the suppression of the interface dipole would shift the CB energy level of TiO_2_ and, consequently, enhance injection process [35].

**Figure 6:**
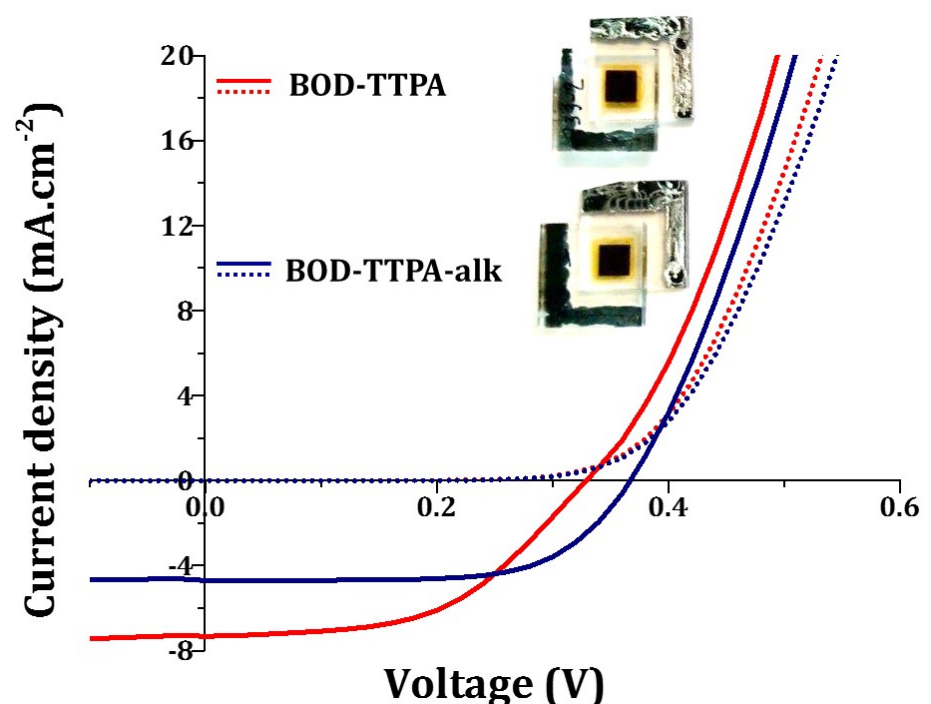
*J*(V) curves of the best performing DSSCs devices sensitized with compounds **BOD-TTPA-alk** (blue traces) and **BOD-TTPA** (red traces) in dark (dashed lines) and under AM1.5G conditions (solid lines). Light source: AM1.5G at 100 mW·cm^−2^, electrolyte composition: 0.5 M of BMII, 0.03 M of I_2_, 0.5 M of LiI, 0.1 M of guanidinium thiocyanate in a mixture of acetonitrile and 3-methoxyproprionitrile (85:15, v/v), electrodes: 12 µm mesoporous anatase TiO_2_ + 4 µm TiO_2_ scattering layer, dyeing bath: [Dye] = 0.2 mM, [CDCA] = 2 mM in CHCl_3_/EtOH 1:1 (v/v)).

The *J*(V) curves of the devices containing compounds **BOD-TTPA-alk** and **BOD-TTPA** are reported in Figure 6. Selected photovoltaic parameters obtained from three different measurements for each dye are gathered in Table 2. **BOD-TTPA-alk** exhibits a maximum power conversion efficiency of 1.12% a short-circuit current (*J*_sc_) of 4.70 mA·cm^−2^, an open-circuit voltage (*V*_oc_) of 0.37 V and a fill factor (FF) of 65%. On the other hand, **BOD-TTPA** displays a power conversion efficiency of 1.22%, along with a *J*_sc_ of 7.33 mA·cm^−2^, a *V*_oc_ of 0.33 V and a FF of 51%. Despite being rather low compared to the performances of more conventional dyes, these results are quite consistent with previous reports on BODIPY sensitizers with absorption in the NIR region [36].

**Table 2:** Photovoltaic parameters of compounds **BOD-TTPA-alk** and **BOD-TTPA** (light source: AM1.5G at 100 mW·cm^−2^, electrolyte composition: 0.5 M of BMII, 0.03 M of I_2_, 0.5 M of LiI, 0.1 M of guanidinium thiocyanate in a mixture of acetonitrile and 3-methoxyproprionitrile (85:15, v/v), electrodes: 12 µm mesoporous anatase TiO_2_, + 4 µm TiO_2_ scattering layer, dyeing bath: [Dye] = 0.2 mM, [CDCA] = 2 mM in CHCl_3_/EtOH 1:1, v/v). Highest value and mean values over three measurements in parenthesis.

Dye	*J*_sc_ (mA·cm^-2^)	*V*_oc_ (V)	FF	PCE (%)

**BOD-TTPA-alk**	4.70 (4.64)	0.37 (0.37)	0.65 (0.64)	1.12 (1.12)
**BOD-TTPA**	7.33 (7.10)	0.33 (0.33)	0.51 (0.50)	1.22 (1.20)

First, comparing the photovoltaic behavior of the two dyes one should note that the *J*_sc_ delivered by the solar cells are rather different (4.70 mA·cm^−2^ for **BOD-TTPA-alk** versus 7.33 mA·cm^−2^ for **BOD-TTPA**). The higher *J*_sc_ obtained with **BOD-TTPA** can be explained with the broader and more intense absorption in the NIR range once grafted on TiO_2_ compared to its alkylated counterpart. Second, one can notice that the *V*_oc_ and FF recorded for devices sensitized with **BOD-TTPA-alk** are slightly higher compared to the ones prepared out of **BOD-TTPA**. This could be explained by the presence of the alkyl chains on the TPA units that are known to prevent the redox mediator to interact with the TiO_2_ surface thus reducing the probability to observe a recombination process between the photo-injected electrons and the iodide.

**Figure 7:**
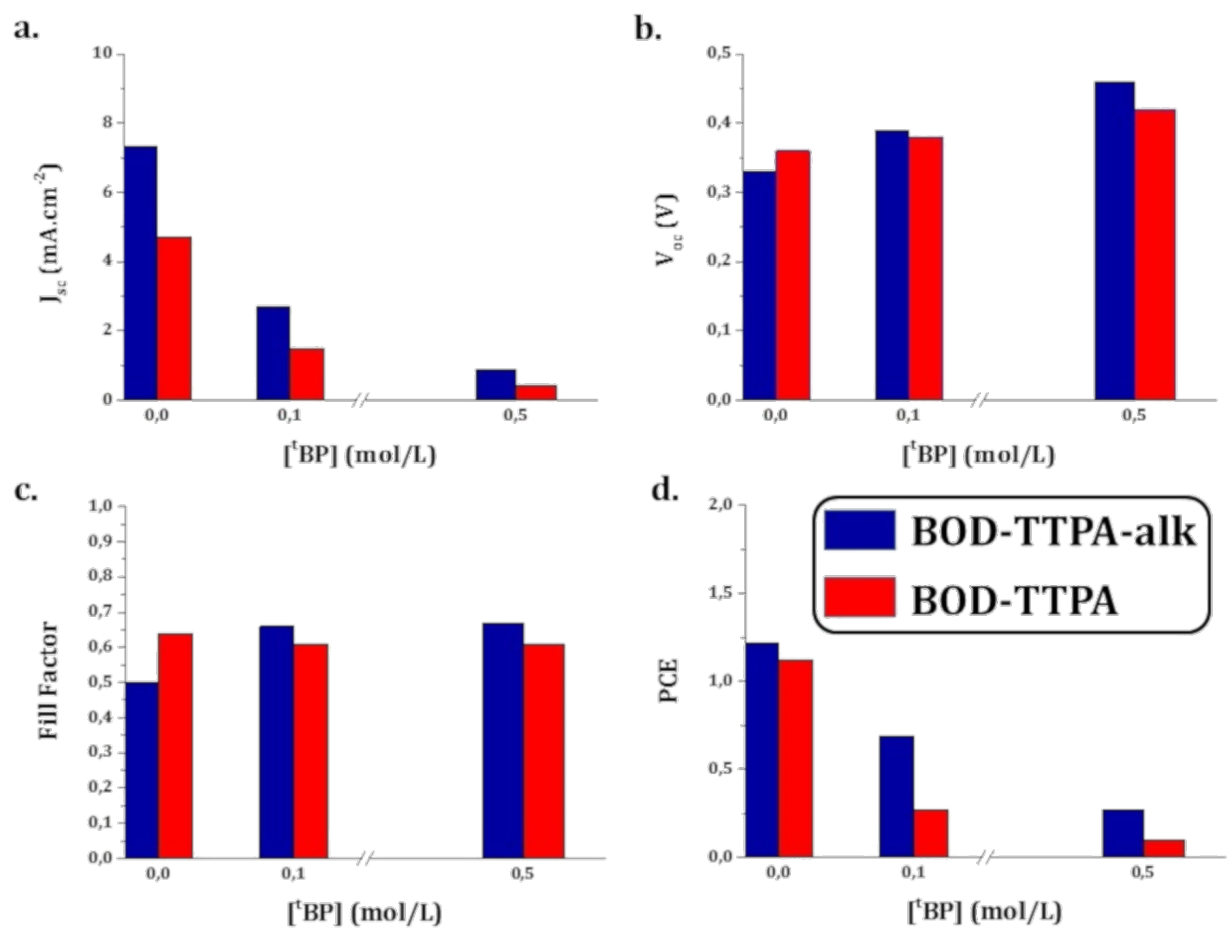
Photovoltaic parameters evolution with the increasing concentration of ^t^BP in the electrolyte.

To confirm that the main factor limiting the device performances is the position of the LUMO level of these dyes with respect to the CB of the oxide, we investigated the effect of ^t^BP content in the electrolyte. By adding ^t^BP in the electrolyte one can tune the electronic properties, i.e., the CB energy level position of TiO_2_ [31,37–39]. The effect is well-known, a negative dipole-related shift of the TiO_2_ Fermi level occurs by ^t^BP adsorption on the surface. As the LUMO levels of the dyes are estimated close to the CB Fermi level, the use of ^t^BP should lead to a drop of the *J*_sc_ because of the reduction of the driving force for the injection. Simultaneously a rise of the *V*_oc_ is expected. A shift of CB energy level toward the vacuum level (i.e., away from the redox potential of the electrolyte) results in a higher open-circuit voltage (*V*_oc_). Indeed, in DSSCs the *V*_oc_ is determined by the quasi-Fermi-level of the metal oxide semiconductor, which is correlated with its CB edge and electron density (see Figure 7).

Solar cells fabricated with various amount of ^t^BP in the electrolyte were investigated (see Table S1 in Supporting Information File 1). It appears from Figure 7 that increasing the concentration of ^t^BP implies, as expected, an increase of *V*_oc_, from 328 mV to 460 mV for **BOD-TTPA-alk** and from 360 mV to 420 mV for **BOD-TTPA**. On the other hand, the *J*_sc_ values for **BOD-TTPA-alk** and **BOD-TTPA** all decrease from 7.33 to 0.88 mA·cm^−2^ and from 4.70 to 0.41 mA·cm^−2^, respectively. This result unambiguously highlights that the shift of TiO_2_ CB impedes the photo-injection of electrons [40]. This proves that the LUMO energy levels of the dyes **BOD-TTPA-alk** and **BOD-TTPA** are too close from the CB of the oxide. Despite a higher *V*_oc_, the loss of *J*_sc_ is responsible for the overall decrease of the power conversion efficiencies.

## Conclusion

We have designed, synthesized and characterized two novel functional BODIPY-based dyes showing intense panchromatic absorption that extends in the NIR region (ε > 2·10^4^ M^−1^·cm^−1^ from 300 to more than 800 nm). The introduction of a cyano-acrylic anchoring function on these dyes allowed us to use them as photosensitizers of TiO_2_ mesoporous electrodes in a DSSC device configuration. We demonstrate PCEs of 1.12% and 1.22%, respectively, for the dye bearing alkyl chains on the TPA unit and for the unsubstituted one. We identified that the limitation of their efficiency in solar cells originates from the unappropriated alignment of their LUMO energy levels with respect to the position of the CB of the metal oxide.

This work highlights that despite remarkable absorption properties, further structural optimization aiming at tuning the LUMO energy level position is necessary. This strategy is very likely to yield more efficient NIR sensitizers. Work following this conclusion is in progress.

## Supporting Information

File 1 Experimental part and copies of NMR spectra.
