# Supplementary material for: Functional panchromatic BODIPY dyes with near-infrared absorption: design, synthesis, characterization and use in dye-sensitized solar cells
Source: Beilstein J Org Chem. 2019 Jul 24;15:1758–68. doi: 10.3762/bjoc.15.169 (PMC6664387; doi:10.3762/bjoc.15.169)
Supplement: File 1 — Experimental part and copies of NMR spectra. [file Beilstein_J_Org_Chem-15-1758-s001.pdf]

BEILSTEIN JOURNAL OF ORGANIC CHEMISTRY

Supporting Information

for

Functional panchromatic BODIPY dyes with near-infrared absorption: design, synthesis, characterization and use in dye-sensitized solar cells

Quentin Huaulmé, Cyril Aumaitre, Outi Vilhelmiina Kontkanen, David Beljonne, Alexandra Sutter, Gilles Ulrich, Renaud Demadrille and Nicolas Leclerc

Beilstein J. Org. Chem. **2019,** *15,* 1758–1768. doi:10.3762/bjoc.15.169

Experimental part and copies of NMR spectra

License and Terms: This is a supporting information file under the terms of the Creative Commons Attribution License (http://creativecommons.org/licenses/by/4.0). Please note that the reuse, redistribution and reproduction in particular requires that the authors and source are credited.
The license is subject to the *Beilstein Journal of Organic Chemistry* terms and conditions: (https://www.beilstein-journals.org/bjoc)

Instrumentation	p. S2
Synthetic procedures	p.S4
NMR spectra	p. S11
HRMS spectra	p. S32
Cyclic voltammetry measurements	p. S34
Devices electrical parameters	p. S35
References	p. S35

Instrumentation

NMR spectroscopy

^{1}H NMR, ^{13}C NMR, ^{11}B NMR and ^{19}F NMR spectra were recorded on a Brucker Avance 400 MHz spectrometer at 298 K if not specified otherwise. ^{1}H and ^{13}C chemical shifts are reported to the relative difference in ppm with respect to the residual deuterated solvent peak: acetone-d6 ($\delta(^1H)$ = 2.06 ppm, $\delta(^{13}C)$ = 206.7 ppm), chloroform-*d* ($\delta(^1H)$ = 7.26 ppm, $\delta(^{13}C)$ = 77.16 ppm), tetrachloroethane-d_2 ($\delta(^1H)$ = 6.00 ppm, $\delta(^{13}C)$ = 73.78 ppm).

Absorption and emission spectroscopies

UV–visible absorption spectra were recorded with a Shimadzu UV-3600 dual-beam grating spectrophotometer using a 1.0 cm quartz cell. The steady-state fluorescence emission and excitation spectra were recorded with a Horiba Jobin Yvon Fluoromax 4P spectrofluorimeter.

Electrochemistry measurements

Oxidation and reduction potentials were determined by cyclic voltammetry with a conventional 3-electrode system using a voltammetric analyzer equipped with a platinum micro disk (2 mm^2) working electrode and a platinum wire counter electrode. The reference electrode is constituted of a non-aqueous silver electrode including the following electrolyte solution: 0.01 M silver nitrate + 0.1 M tetrabutylammonium perchlorate in acetonitrile. Potentials were calibrated versus the saturated calomel electrode (SCE), using the ferrocene/ferrocinium (Fc/Fc^+) couple as an internal reference and a conventional scan rate of 150 mV/s. Recrystallized tetrabutylammonium hexafluorophosphate (Bu_4NPF_6) was used as the supporting electrolyte (0.1 M) in distilled and anhydrous DCM. All potentials are referred to the SCE electrode that was calibrated at 0.38 V vs the Fc/Fc^+ system. Following the work of Jenekhe et al.,[1] we estimated the electron affinity (EA) or lowest unoccupied molecular orbital (LUMO) and the ionization potential (IP) or highest occupied molecular orbital (HOMO) from the redox data. The LUMO and HOMO levels were calculated from the following equations, using the oxidation and reduction onset potentials: LUMO (eV) = −[E_{onset}red (vs SCE) + 4.4] and HOMO (eV) = −[E_{onset}ox (vs SCE) + 4.4], based on an SCE energy level of 4.4 eV relative to the vacuum.

DSSC devices elaboration

12 µm thick opaque anatase TiO_2 electrodes are washed with absolute ethanol and dried with argon. $TiCl_4$ (0.45 mL, 778 mg, 4.10 mmol) is added dropwise to water (100 mL) at 4 °C. The solution is further stirred 5 minutes in an ice-cooled water. The titanium electrodes are placed in the above-mentioned solution at 70 °C for 20 min. At room temperature, the treated electrodes are rinsed with water, absolute ethanol, and are then dried with an argon flux. They are then calcinated under air at 500 °C for 20min. Once cooled down to room temperature, the electrodes are immerged in the dyeing bath overnight ([Dye] = 0.50 M, [CDCA] = 5.0 M in a mixture of

chloroform and *tert*-butanol (1:1, v/v)). After 16 hours of impregnation, the photoelectrodes are successively rinsed with absolute EtOH and chloroform, and then dried under an argon flux. The counter electrodes are washed with ethanol and dried under argon flux. They are coated with a platisol solution (Solaronix, Switzerland), and then charred at 500°C under air for 20 minutes. The photoanode and the counterelectrode are sealed using a thermosealing polymer (60 thick, Solaronix, Swtizerland). The resulting device is filled with the appropriate electrolyte. Note that the devices are realized **one by one** (so that the exposure to the air is reduced).

Synthetic procedures

Compound 1

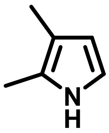

To a solution of 2-butanone (15.0 g, 208 mmol, 1.00 equiv) and hydroxylamine hydrochloride (28.9 g, 416 mmol, 2.00 equiv) in DMSO (100 mL) is carefully and portionwise added $NaHCO_3$ (31.4 g, 374 mmol, 1.80 equiv). The mixture is stirred at 60 °C overnight (18 h). At rt, the reaction mixture is portioned between brine (100 mL) and Et_2O (100 mL). The aqueous layer is extracted twice with Et_2O (2 × 50 mL). The organic layer is concentrated under vacuum. The hydroxylamine intermediate is solubilized in anhydrous DMSO (100 mL). The mixture is degassed by gentle bubbling of acetylene at R.T. for 15 minutes. KOH (17.5 g, 312 mmol, 1.50 equiv) is added and the reaction mixture is stirred at 110 °C for 5 h, with continuous gentle bubbling of acetylene (ca. 15 $cm^3 \cdot min^{-1}$). At rt, the reaction mixture is portioned between water (150 mL) and Et_2O (100 mL). The aqueous layer is extracted twice with Et_2O (2 × 50 mL). The combined organic layer is dried over $MgSO_4$, filtered off and concentrated under vacuum. The resulting black residue is purified by column chromatography (Al_2O_3, PE/Et2O 3:1) to afford pure compound 1 as a colorless oil that darkens slowly at rt (4.88 g, 51.3 mmol, 24% over two steps). Analytical data were found to be in accordance with the relevant literature. [2] **^{1}H-NMR (Acetone-*d*$_6$, 298 K, 400 MHz) δ (ppm):** 9.37 (bs, 1H), 6.47 (d, J = 2.6 Hz, 1H), 5.79 (d, J = 2.6 Hz, 1H), 2.10 (s, 3H), 1.94 (s, 3H). **^{13}C-NMR (Acetone-d$_6$, 298 K, 100 MHz) δ (ppm):** 124.0, 115.7, 114.1, 110.4, 11.5, 11.2.

Compound 2

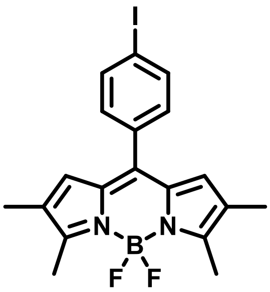

A solution of 2,3-dimethylpyrrole (2.00 g, 21.0 mmol, 2.50 equiv) in anhydrous DCM (200 mL) is degassed with Argon for 10 minutes. 4-Iodobenzoyl chloride (2.24 g, 8.41 g, 1.00 equiv) is added in one go and the mixture is stirred at RT for 5 days. Anhydrous NEt_3 (6.81 g, 67.3 mmol, 8.00 equiv) and $BF_3.OEt_2$ (7.15 g, 50.4 mmol, 6.00 equiv) are carefully added at 0 °C. The reaction mixture is allowed to warm up to rt and is stirred at this temperature for 2 additional hours. The mixture is poured on saturated $NaHCO_3$ aqueous solution (100 mL). The aqueous layer is extracted with DCM (2 × 50 mL). The combined organic layer is dried over $MgSO_4$, filtered off and concentrated to dryness. The resulting black residue is filtered through a pad of silica (DCM/PE 1/1). The dark red fraction is collected and concentrated to dryness. Final purification is achieved by column chromatography (PE/DCM 7/3) to afford the pure expected compound as a red solid (1.32 g, 2.94 mmol, 35%). **^{1}H-NMR (CDCl$_3$, 298 K, 400 MHz) δ (ppm):** 7.81 (d, ^{3}J = 8.4 Hz, 2H), 7.20 (d, J= 8.4 Hz, 2H), 6.42 (bs, 2H), 2.54 (s, 6H), 1.99 (d, ^{4}J = 0.77 Hz, 6H). **^{13}C-NMR (CDCl$_3$, 298 K, 100 MHz) δ (ppm):** 156.3, 138.7, 137.5, 134.1, 132.6, 132.0, 128.3, 12.8, 11.2.

Compound 3

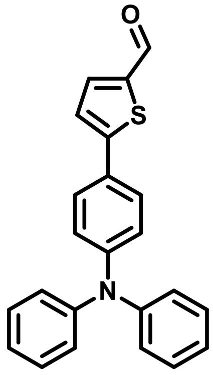

A solution of 5-bromothiophene-2-carbaldehyde (702 mg, 3.67 mmol, 1.00 equiv) and (4-(diphenylamino)phenyl)boronic acid (1.16 g, 4.04 mmol, 1.10 equiv) in a mixture of aqueous 1 M K_2CO_3 (7.4 mL, 2.0 equiv) and toluene (50 mL) is degassed with Argon. The reaction mixture is stirred at 100 °C overnight (18 h). At rt, the mixture is portioned between water (40 mL) and Et_2O (40 mL). The aqueous layer is extracted twice with Et_2O (2 × 50 mL). The combined organic layer is washed with brine, dried over $MgSO_4$, filtered off and concentrated to dryness. The organic layer is concentrated under vacuum. The resulting residue is purified by column chromatography (PE/DCM) to afford the expected compound **3** as a colorless oil (1.04 g, 2.93 mmol, 80%). Analytical data were found to be in accordance with the relevant literature. [3] **^{1}H-NMR ($CDCl_3$, 298 K, 400 MHz) δ (ppm):** 9.85 (s, 1H), 7.71 (d, J = 4.0 Hz, 1H), 7.52 (d, J = 8.0 Hz, 2H), 7.28-7.32 (m, 5H), 7.05-7.15 (m, 8H). **^{13}C-NMR ($CDCl_3$, 298 K, 100 MHz) δ (ppm):** 182.7, 154.7, 149.3, 147.1, 141.5, 137.9, 129.6, 127.4, 126.3, 125.3, 124.0, 123.0, 122.5.

Compound 4

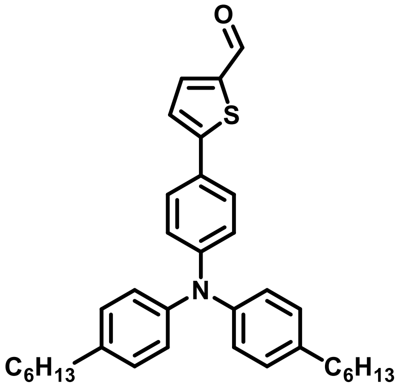

To a solution of 4-bromo-*N*,*N*-bis(4-hexylphenyl)aniline (1.50 g, 3.04 mmol, 1.00 equiv) in anhydrous THF (15 mL) is added dropwise at −78 °C *n*-BuLi (2.5 M in hexanes, 1.3 mL, 3.3 mmol, 1.1 equiv). The reaction mixture is stirred at −78 °C for 30 minutes before trimethyltin chloride (1.0 M in Et_2O, 3.4 mL, 3.4 mmol, 1.1 equiv) is added in one portion. The mixture is allowed to warm up to rt overnight (18 h). Water (50 mL) and Et_2O (70 mL) are successively added. The aqueous layer is extracted once with Et_2O (50 mL). The combined organic layer is washed with brine, dried over $MgSO_4$, filtered off and concentrated to dryness. The organic layer is concentrated under vacuum. The resulting yellow oil is used without any further purification.

A solution of 5-bromothiophene-2-carbaldehyde (401 mg, 2.10 mmol, 1.00 equiv) and the previous organotin intermediate in anhydrous toluene (60 mL) is degassed for 20 minutes with argon. $Pd_2(dba)_3$ (57 mg, 0.063 mmol, 3 mol %) and P(*o*-tolyl)$_3$ (76 mg, 0.25 mmol, 12 mol %) are successively added and the reaction mixture is stirred at 110 °C overnight (18 h). At rt, the reaction mixture is concentrated under vacuum (extra precaution should be taken to avoid any contamination with $BrSnMe_3$). The residue is purified by column chromatography (PE/EtOAc 4:1) to afford pure compound **4** as a pale yellow solid (527 mg, 1.01 mmol, 48 %). **^{1}H-NMR ($CDCl_3$, 298 K, 400 MHz) δ (ppm):** 9.84 (s, 1H), 7.69 (d, ^{3}J = 4.0 Hz, 1H), 7.48 (d, ^{3}J = 8.9 Hz, 2H), 7.27 (d, 4.0 Hz, 1H), 6.99-7.11 (m, 10H), 2.58 (t, J = 8.0 Hz, 4H), 1.58-1.65 (m, 4H), 1.31-1.34 (m, 12H), 0.90 (m, 6H). **^{13}C-NMR ($CDCl_3$, 298 K, 100 MHz) δ (ppm):** 182.7, 155.1, 149.7, 144.6, 141.2, 139.0, 138.0, 129.5, 127.8, 125.5, 152.3, 122.7, 121.3, 35.6, 31.9, 31.6, 29.2, 22.8, 14.2.

Compound 5

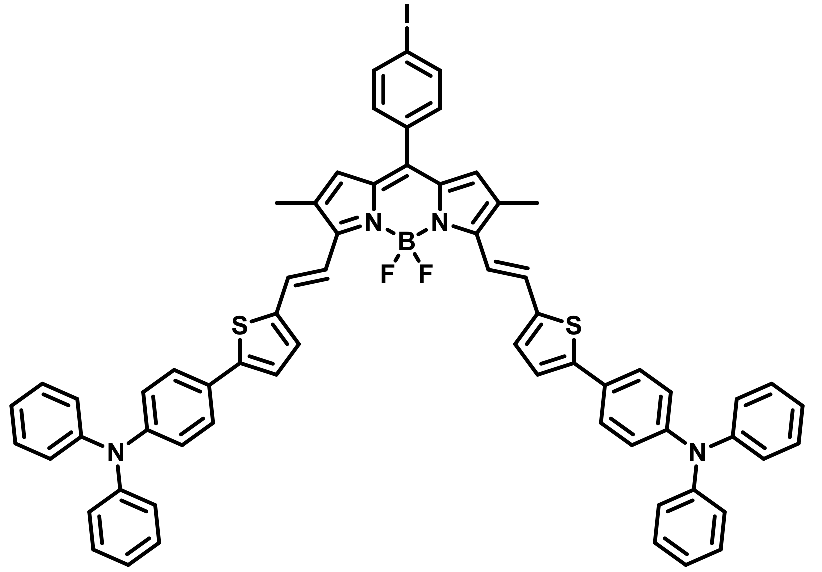

To a solution of compound **2** (120 mg, 0.266 mmol, 1.0 equiv) and compound **3** (236 mg, 0.665 mmol, 2.50 equiv) in toluene (5 mL) and piperidine (1 mL) is added a catalytic amount of PTSA (<2 mg). The mixture is heated under reflux for 20 minutes. The solvents are removed under vacuum. The residue is purified by column chromatography (cyclohexane /toluene/DCM 5:3:2) to afford pure compound **5** as a black solid (104 mg, 0.0924 mmol, 35 %). **^{1}H-NMR ($CDCl_3$, 298 K, 400 MHz) δ (ppm):** 7.83 (d, ^3J = 8.3 Hz, 2H), 7.58 (d, ^3J = 16.3 Hz, 2H), 7.52 (d, ^3J = 8.7 Hz, 4H), 7.44 (d, ^3J = 16.3 Hz, 2H), 7.03-7.29 (m, 30H), 6.48 (s, 2H), 2.32 (s, 6H). **^{13}C-NMR ($CDCl_3$, 298 K, 100 MHz) δ (ppm):** 151.4, 147.7, 147.3, 146.1, 141.6, 137.4, 134.9, 134.4, 132.1, 130.3, 129.6, 129.4, 128.8, 127.9, 126.7, 124.7, 123.4, 123.3, 118.7, 95.6, 14.9.

Compound 6

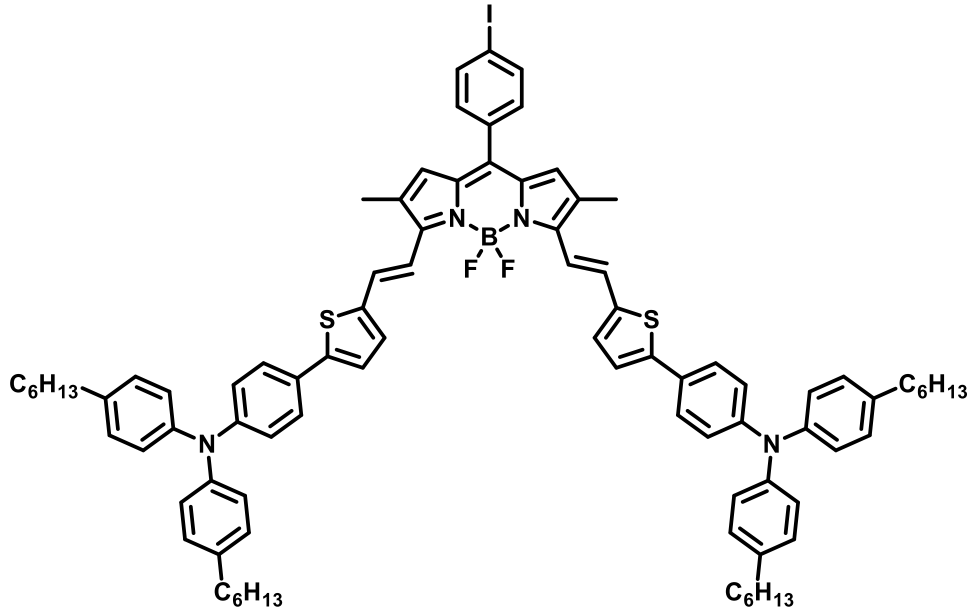

To a solution of compound **2** (120 mg, 0.266 mmol, 1.00 equiv) and compound **4** (325 mg, 0.622 mmol, 2.50 equiv) in toluene (5 mL) and piperidine (1 mL) is added a catalytic amount of PTSA (<2 mg). The mixture is heated at reflux for 20 minutes. The solvent are removed under vacuum. The residue is purified by column chromatography (PE/DCM 3:2). Final purification is achieved by recrystallization from DCM/EtOH to afford pure compound **5** as a black solid (120 mg, 0.0821 mmol, 30 %). **^{1}H-NMR ($CDCl_3$, 298 K, 400 MHz) δ (ppm):** 7.82 (d, ^3J =8.1 Hz, 2H), 7.57 (d, ^3J =16.1 Hz, 2H), 7.48 (d, ^3J =7.5 Hz, 4H), 7.44 (d, ^3J =16.1 Hz, 2H),,7.18-7.27 (m, 6H), 7.01-7.09 (m, 20H), 6.47 (s, 2H), 2.57 (t, ^3J =8.0 Hz; 8H), 2.32 (s, 6H), 1.57-1.65 (m, 8H), 1.21-1.41 (m, 24H), 0.88-0.91 (m, 12H). **^{13}C-NMR ($CDCl_3$, 298 K, 100 MHz) δ (ppm):** 148.3, 146.6, 145.1, 141.4, 138.3, 137.6, 135.0, 134.5, 132.2, 130.4, 129.9, 126.6, 125.0, 123.2, 122.3, 95.7, 35.6, 31.9, 31.6, 29.2, 22.8, 15.0, 14.3.

Compound 7

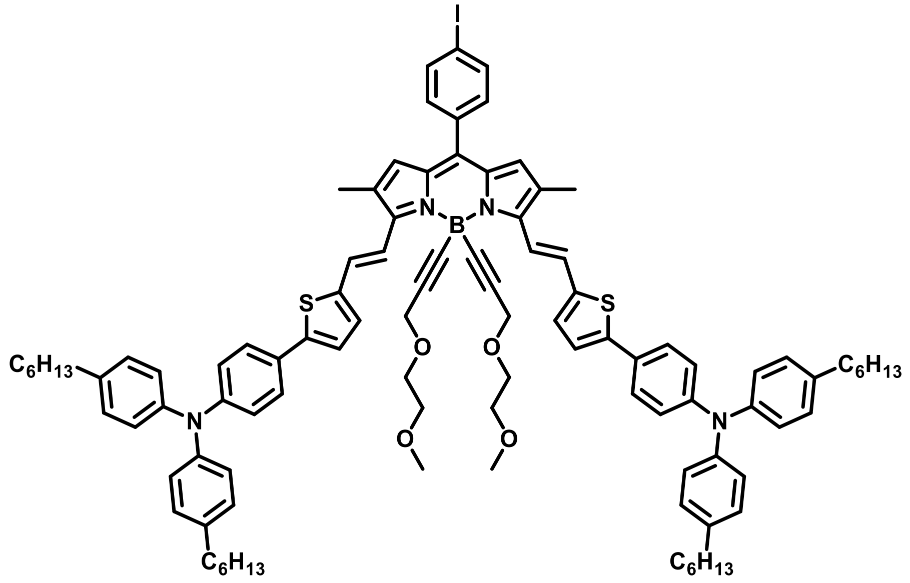

To a solution of 3-(2-methoxyethoxy)prop-1-yne (84 mg, 0.74 mmol, 3.0 equiv) in anhydrous THF (5.0 mL) is added at rt EtMgBr (1.0 M in THF, 0.66 mmol, 2.7 equiv). The mixture is stirred at 60 °C for 2 hours. At rt, the resulting Grignard intermediate is transferred via cannula on a solution of compound **5** (360 mg, 0.246 mmol, 1.00 equiv) in anhydrous THF (10 mL). The reaction mixture is stirred at 60 °C overnight (20 h). At rt, the reaction mixture is portioned between saturated $NaHCO_3$ aqueous solution (50 mL) and Et_2O (100 mL). The aqueous layer is extracted twice with Et_2O (2 × 50 mL). At rt, the combined organic layer is dried over $MgSO_4$, filtered off and concentrated to dryness. The resulting residue is purified by column chromatography to afford pure compound **7** as a black solid (344 mg, 0.209 mmol, 85 %). **^{1}H-NMR ($CDCl_3$, 298 K, 400 MHz) δ (ppm):** 8.17 (d, ^3J = 16.3 Hz, 2H), 7.82 (d, ^3J =8.3 Hz, 2H), 7.47 (d, ^3J = 8.8 Hz, 4H), 7.29 (d, ^3J = 16.3 Hz, 2H), 7.22-7.24 (m, 4H), 7.19 (d, ^3J =3.8 Hz, 2H), 7.01-7.09 (m, 20H), 6.48 (s, 2H), 4.22 (s, 4H), 3.64-3.67 (m, 4H), 3.27-3.30 (m, 4H), 3.14 (s, 6H), 2.55 (t, ^3J =7.9 Hz, 8H), 2.34 (s, 6H), 1.57-1.64 (m, 8H), 1.27-1.38 (m, 24H), 0.89 (t, ^3J = 6.7 Hz, 12H). **^{13}C-NMR ($CDCl_3$, 298 K, 100 MHz) δ (ppm)**: 150.7, 148.4, 146.1, 145.0, 141.9, 138.4, 137.4, 133.0, 132.3, 130.3, 129.4, 128.8, 127.8, 127.0, 126.6, 125.0, 123.3, 122.2, 121.2, 102.1, 95.6, 71.8, 68.4, 59.6, 58.9, 35.6, 31.9, 31.6, 29.2, 22.8, 15.5, 14.3.

Compound 8

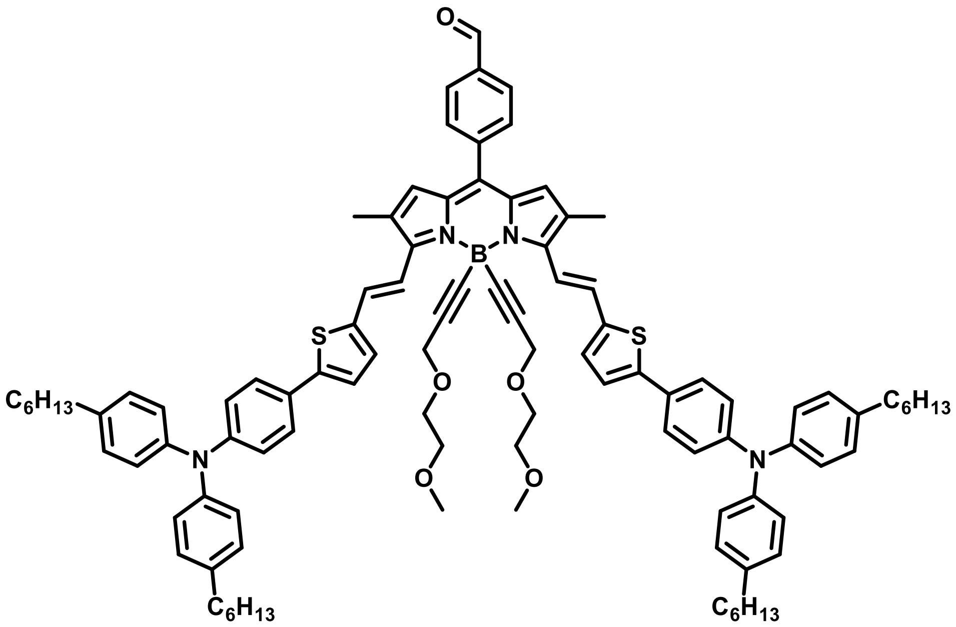

A solution of compound **7** (117 mg, 0.0714 mmol, 1.00 equiv) and sodium formiate (29 mg, 0.42 mmol, 6.0 equiv) in anhydrous DMF (5.0 mL) is degassed with a CO stream bubbling in the solution at 60 °C for 10 minutes. $Pd(PPh_3)_2Cl_2$ (5 mg, 0.0071 mmol, 10 mol%) is then added and the reaction mixture is stirred at 100 °C under CO atmosphere for 6 hours. At rt, the reaction mixture is portioned between water (30 mL) and EtOAc (50 mL). The aqueous layer is extracted twice with EtOAc (2 × 15 mL). At rt, the combined organic layer is dried over $MgSO_4$, filtered off and concentrated to dryness. The resulting residue is purified by column chromatography (Al_2O_3, DCM/PE 1:1) to afford pure compound **8** as a dark brown solid (21.7 mg, 0.0139 mmol, 19%). **^{1}H-NMR (CD_2Cl_2, 298 K, 400 MHz) δ (ppm):** 10.11 (s, 1H), 8.14 (d, ^3J = 16.3 Hz, 2H), 7.99 (d, ^3J =8.2 Hz, 2H), 7.70 (d, ^3J = 8.1 Hz, 2H), 7.49 (d, ^3J = 8.7 Hz, 4H), 7.37 (d, ^3J = 16.3 Hz, 2H), 7.25 (d, ^3J = 3.9 Hz, 2H), 7.22 (d, ^3J = 3.9 Hz, 2H), 7.08 (d, ^3J = 8.4 Hz, 8H), 7.01 (d, ^3J = 8.4 Hz, 8H), 6.98 (d, ^3J = 8.7 Hz, 4H), 6.52 (s, 2H), 4.14 (s, 4H), 3.56-3.58 (m, 4H), 3.24-3.26 (m, 4H), 3.08 (s, 6H), 2.55 (t, ^3J = 8.0 Hz ; 8H), 2.36 (s, 6H), 1.55-1.61 (m, 8H), 1.28-1.37 (m, 24H), 0.87 (t; ^3J = 6.9 Hz, 12H). **^{13}C-NMR (CD_2Cl_2, 298 K, 100 MHz) δ (ppm)**: 192.0, 151.1, 148.8, 146.6, 145.2, 142.1, 141.3, 138.9, 137.2, 134.8, 133.2, 131.7, 131.0, 129.9, 129.7, 129.6, 128.7, 128.0, 127.0, 126.8, 125.4, 123.6, 122.1, 121.1, 92.8, 72.0, 68.9, 59.6, 58.8, 35.6, 32.2, 32.0, 29.5, 23.0, 15.5, 14.3.

Compound BOD-TTPA-alk

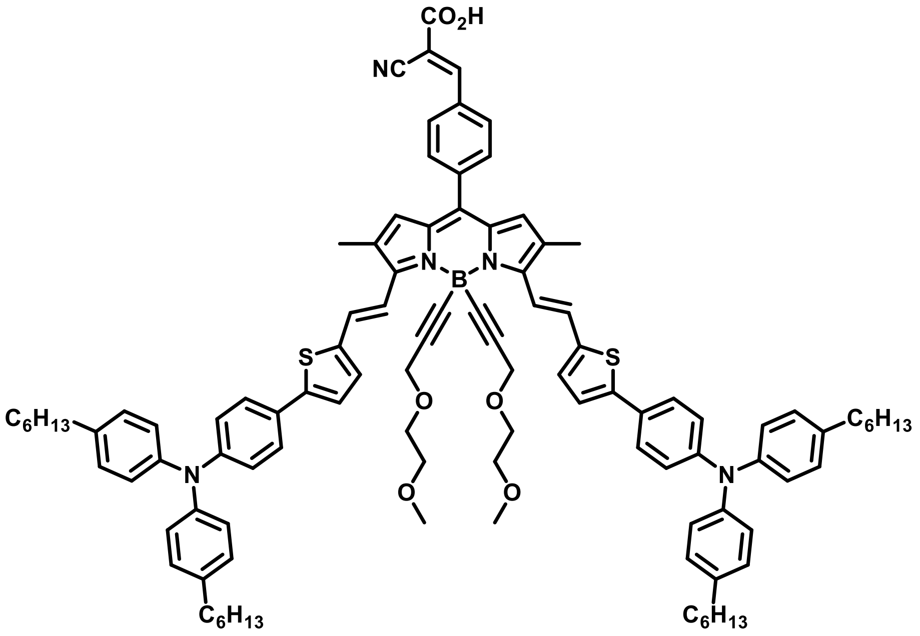

To a solution of compound **8** (36.9 mg, 0.0238 mmol, 1.00 equiv) in ACN and chloroform (6.0 mL and 2.0 mL, respectively) are successively added piperidine (0.5 mL) and cyanoacetic acid (20 mg, 0.238 mmol, 10 equiv). The reaction mixture is stirred at 70 °C overnight (18 h). At rt, the reaction mixture is portioned between water (30 mL) and EtOAc (50 mL). The aqueous layer is extracted twice with EtOAc (2 × 15 mL). At rt, the combined organic layer is dried over $MgSO_4$, filtered off and concentrated to dryness. The resulting residue is purified by column chromatography (SiO_2, DCM/EtOH 95:5 to DCM/EtOAc/EtOH 1:1:2) to afford **BOD-TTPA-alk** as a dark brown solid (10 mg, 0.00617 mmol, 26 %). **^{1}H-NMR ($CDCl_3$, 298 K, 400 MHz) δ (ppm):** 8.14 (d, J = 1.4 Hz,, 2H), 7.61–7.48 (bs, 1H), 7.34-7.47 (m, 4H), 7.13-7.21 (m, 2H); 6.83-7.09 (m, 28 H), 6.30-6.49 (bs, 2H), 4.07-4.30 (bs, 4H), 3.57-3.72 (bs, 4H), 3.19-3.30 (bs, 4H), 3.02-3.14 (bs, 6H), 2.47-2.58 (m, 8H), 1.53-1.63 (m, 6H), 1.17-1.37 (m, 42 H). **HRMS (ESI-TOF):** Calculated for $C_{105}H_{116}BN_5O_6S_2$: 1617.8460, found $[M+H]^+$ 1618.8495.

Compound 9

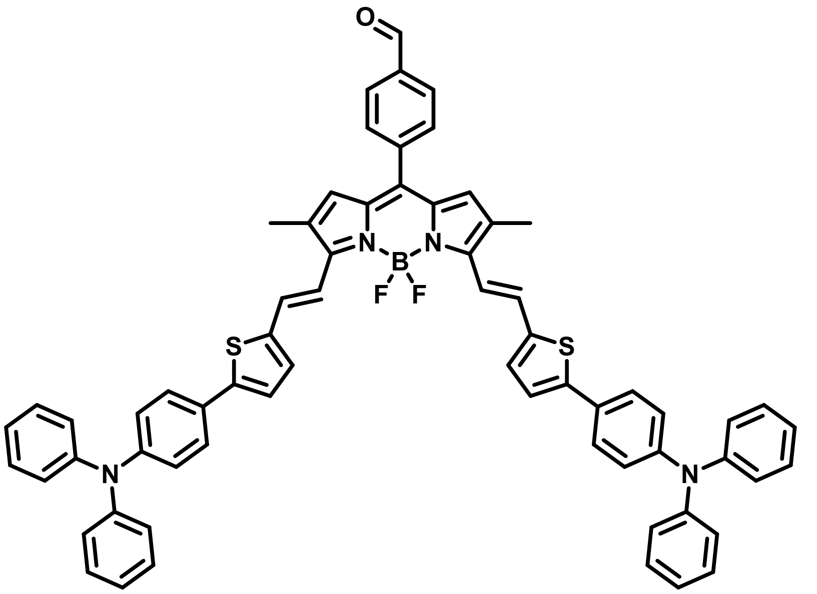

A solution of compound **5** (100 mg, 0.0896 mmol, 1.00 equiv) and sodium formiate (37 mg, 0.53 mmol, 6.0 equiv) in anhydrous DMF (5.0 mL) is degassed with a CO stream bubbling in the solution at 60 °C for 10 minutes. $Pd(PPh_3)_2Cl_2$ (6.0 mg, 0.0, 10 mol %) is then added and the reaction mixture is stirred at 100 °C under CO atmosphere for 6 hours. At rt, the reaction mixture is portioned between water (30 mL) and EtOAc (50 mL). The aqueous layer is extracted twice with EtOAc (2 × 15 mL). At rt, the combined organic layer is dried over $MgSO_4$, filtered off and concentrated to dryness. The resulting residue is purified by column chromatography (PE/DCM) to afford pure compound **9** as a dark brown solid (33 mg, 0.032 mmol, 35%).**^{1}H-NMR (CD_2Cl_2, 298 K, 400 MHz) δ (ppm):** 10.10 (s, 1H), 7.99 (d, ^3J = 7.9 Hz, 2H), 7.68 (d, ^3J = 8.0 Hz, 2H), 7.50-7.54 (m, 8H), 1.24-7.29 (m, 12H, 7.02-7.11 (m, 16H), 6.50 (s, 2H), 2.33 (s, 6H). **^{13}C-NMR ($CDCl_3$, 298 K, 100 MHz) δ (ppm):** 191.7, 165.4, 165.0, 151.6, 147.8, 147.3, 146.3, 141.5, 141.0, 134.9, 131.1, 130.0, 139.8, 129.7, 129.4, 129.3, 128.7, 127.8, 126.7, 124.7, 123.3, 123.3, 118.6, 14.9.

Compound BOD-TTPA

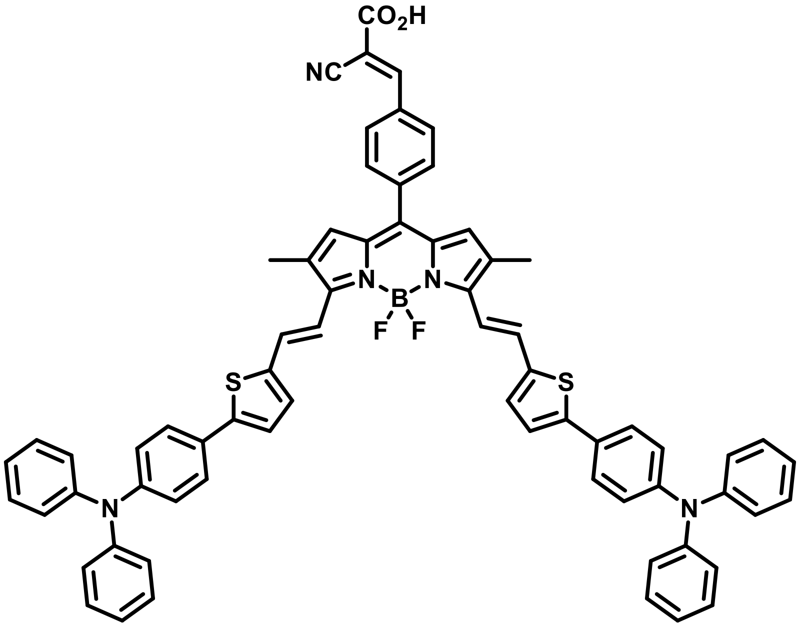

To a solution of compound **10** (33 mg, 0.0322 mmol, 1.00 equiv) in ACN and chloroform (6.0 mL and 2.0 mL respectively) are successively added piperidine (0.5 mL) and cyanoacetic acid (2.7 mg, 0.0322 mmol, 1.00 equiv). The reaction mixture is stirred at 70 °C overnight (18 h). At rt, the reaction mixture is portioned between water (30 mL) and EtOAc (50 mL). The aqueous layer is extracted twice with EtOAc (2 × 15 mL). The combined organic layer is dried over $MgSO_4$, filtered off and concentrated to dryness. The resulting residue is purified by column chromatography (SiO_2, DCM/EtOH 95:5 to DCM/EtOAc/EtOH 1:1:2) to afford pure compound **9** as a dark brown solid (12 mg, 0.00617 mmol, 34%). **^{1}H-NMR ($CDCl_3$, 298 K, 400 MHz) δ (ppm):**11.65 (bs, 1H), 7.58 (s, 1H), 7.37-7.40 (m, 3H), 6.93 (d, ^3J = 8.3 Hz, 2H), 6.76-6.86 (m, 8H), 6.55-6.61 (m, 11H), 6.35-6.46 (m, 16 H), 5.88 (s, 2H), 1.55 (s, 6H). **^{13}C-NMR ($CDCl_3$, 298 K, 100 MHz) δ (ppm):** 178.6, 150.7, 150.1, 147.2, 146.5, 145.7, 140.8, 134.3, 133.0, 130.6, 130.1, 129.6, 129.4, 129.2, 128.9, 126.8, 126.1, 124.2, 122.9, 122.8, 122.6, 117.9, 117.5, 14.4. **HRMS (ESI-TOF):** Calculated for $C_{69}H_{50}BF_2N_5O_2S_2$: 1093.3473, found $[M]^+$: 1093.3450.

NMR spectra

Figure S1: ^{1}H–NMR of compound 1 (acetone-d_6, 400 MHz, 298 K)

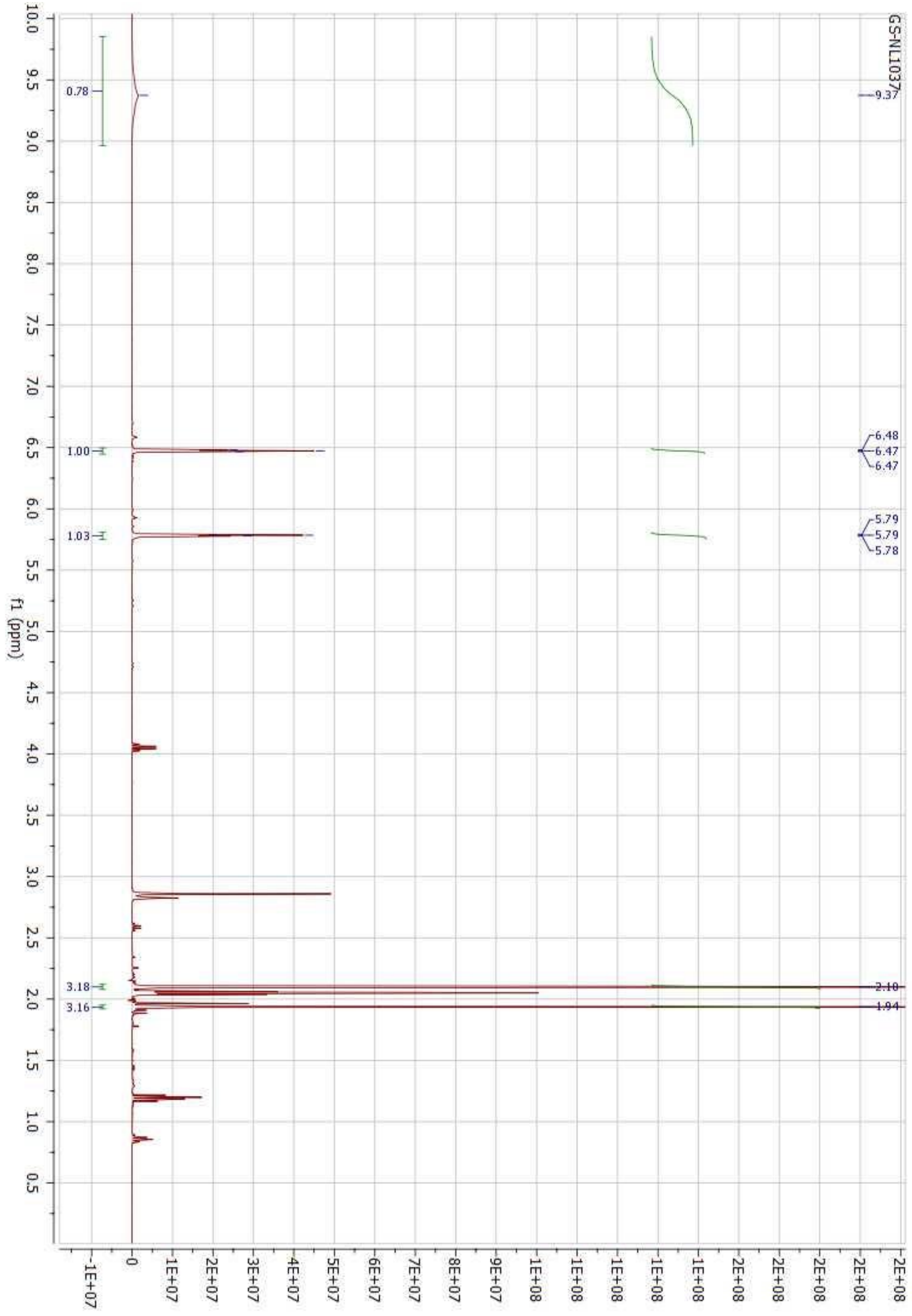

Figure S2: ^{13}C–NMR of compound 1 (acetone-d_6, 100 MHz, 298 K)

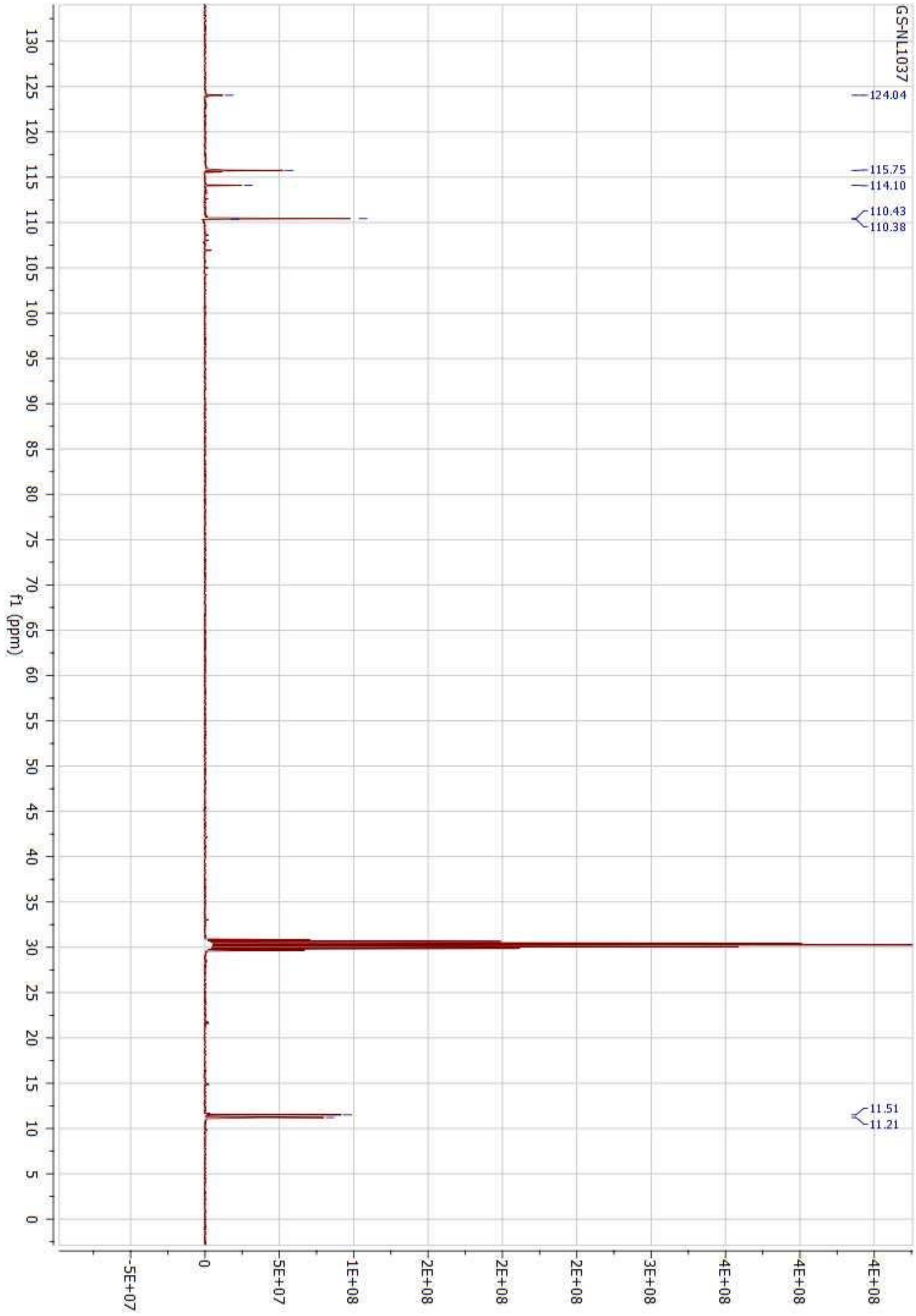

Figure S3: ^{1}H–NMR of compound 2 ($CDCl_3$, 400 MHz, 298 K)

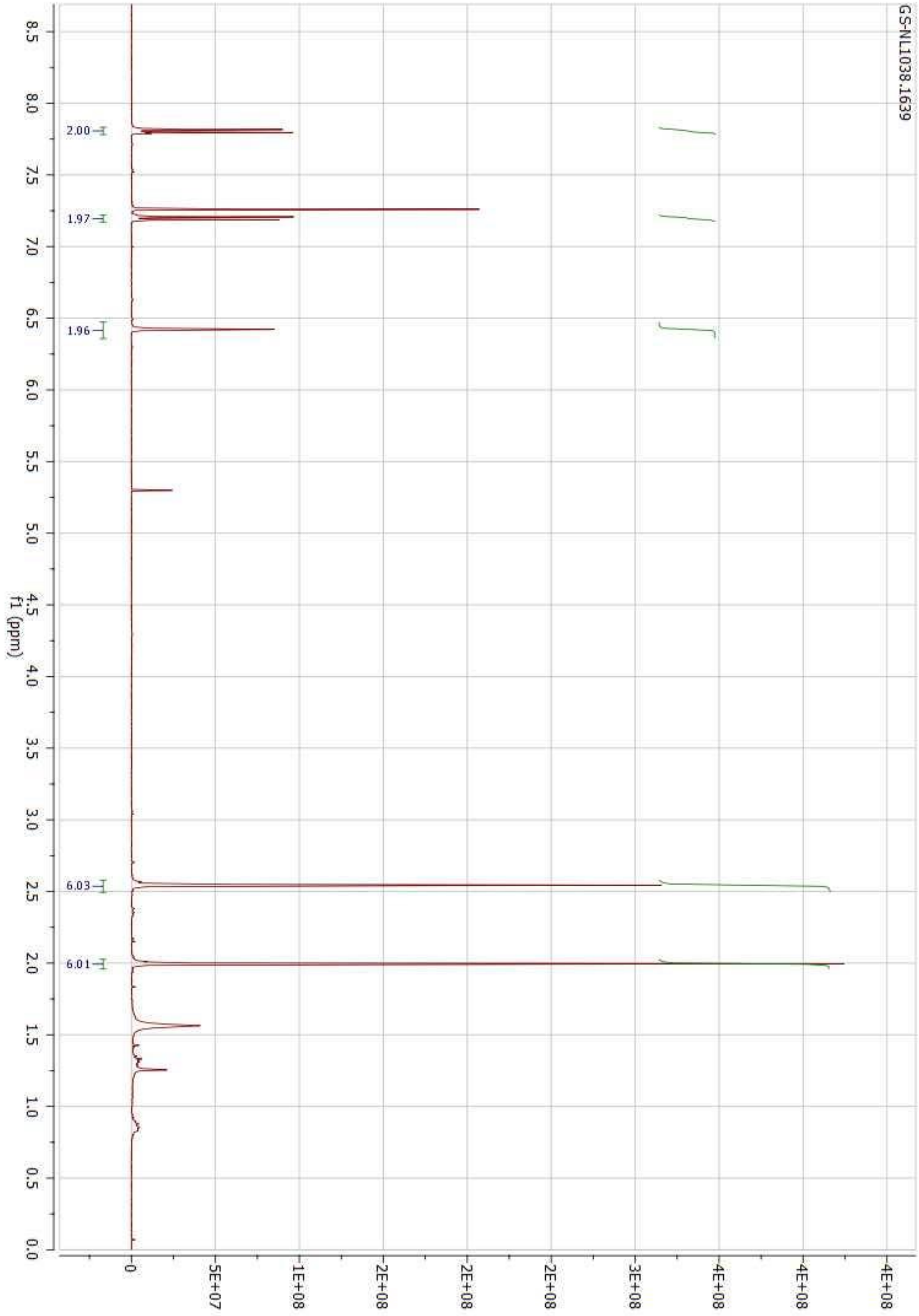

Figure S4: ^{13}C–NMR of compound 2 ($CDCl_3$, 100 MHz, 298 K)

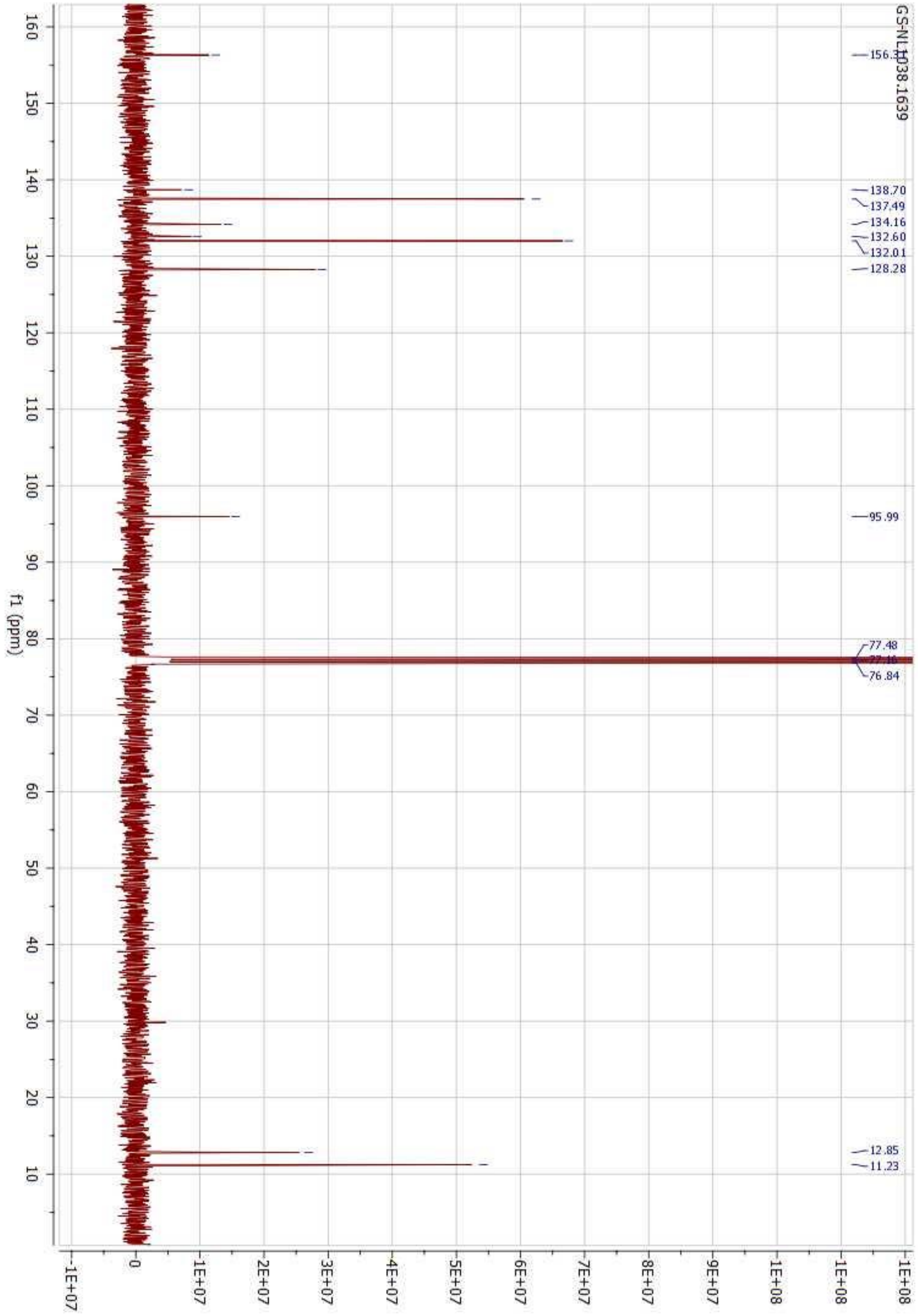

Figure S5: ^{1}H–NMR of compound 3 ($CDCl_3$, 400 MHz, 298 K)

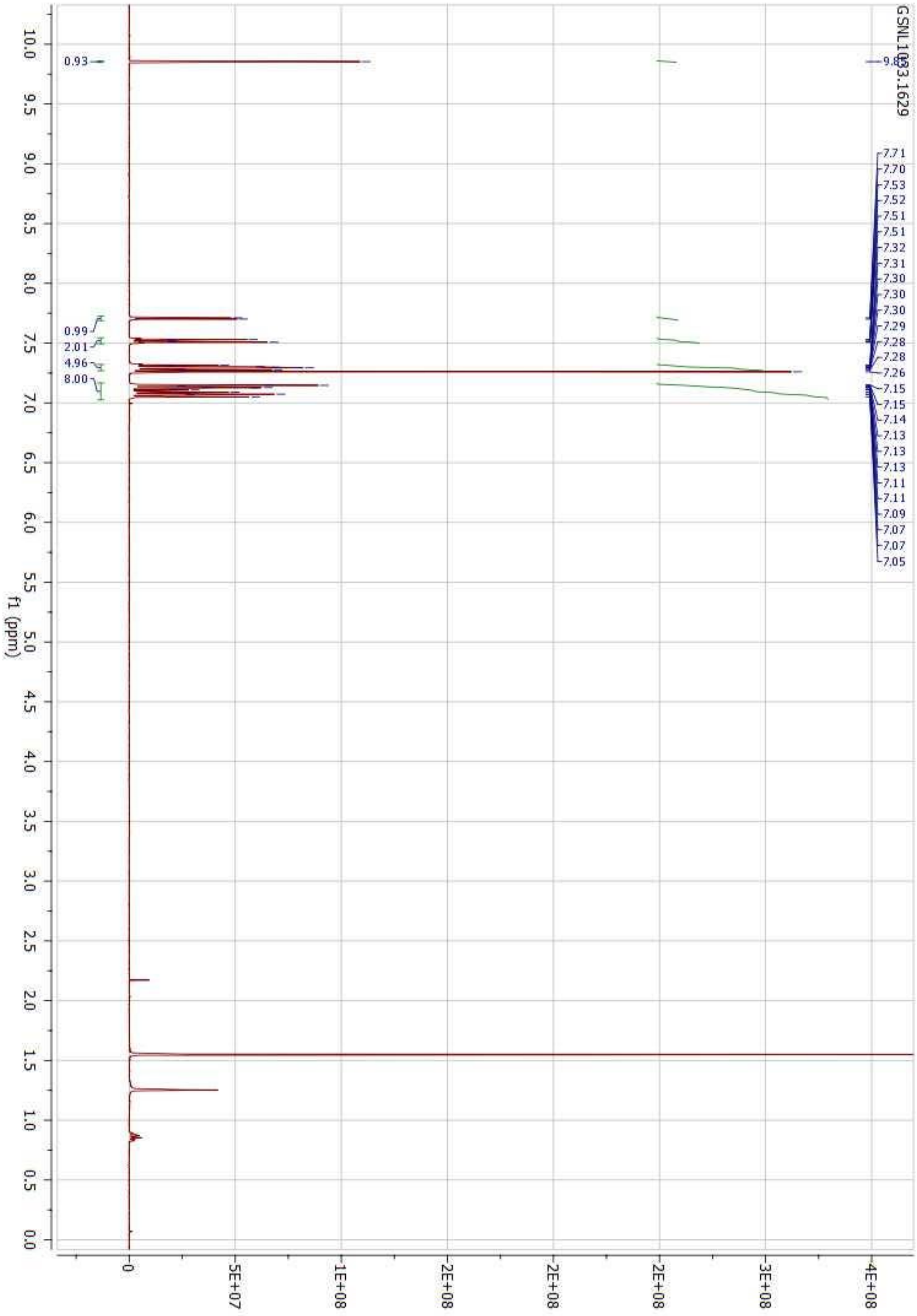

Figure S6: ^{13}C–NMR of compound 3 ($CDCl_3$, 100 MHz, 298 K)

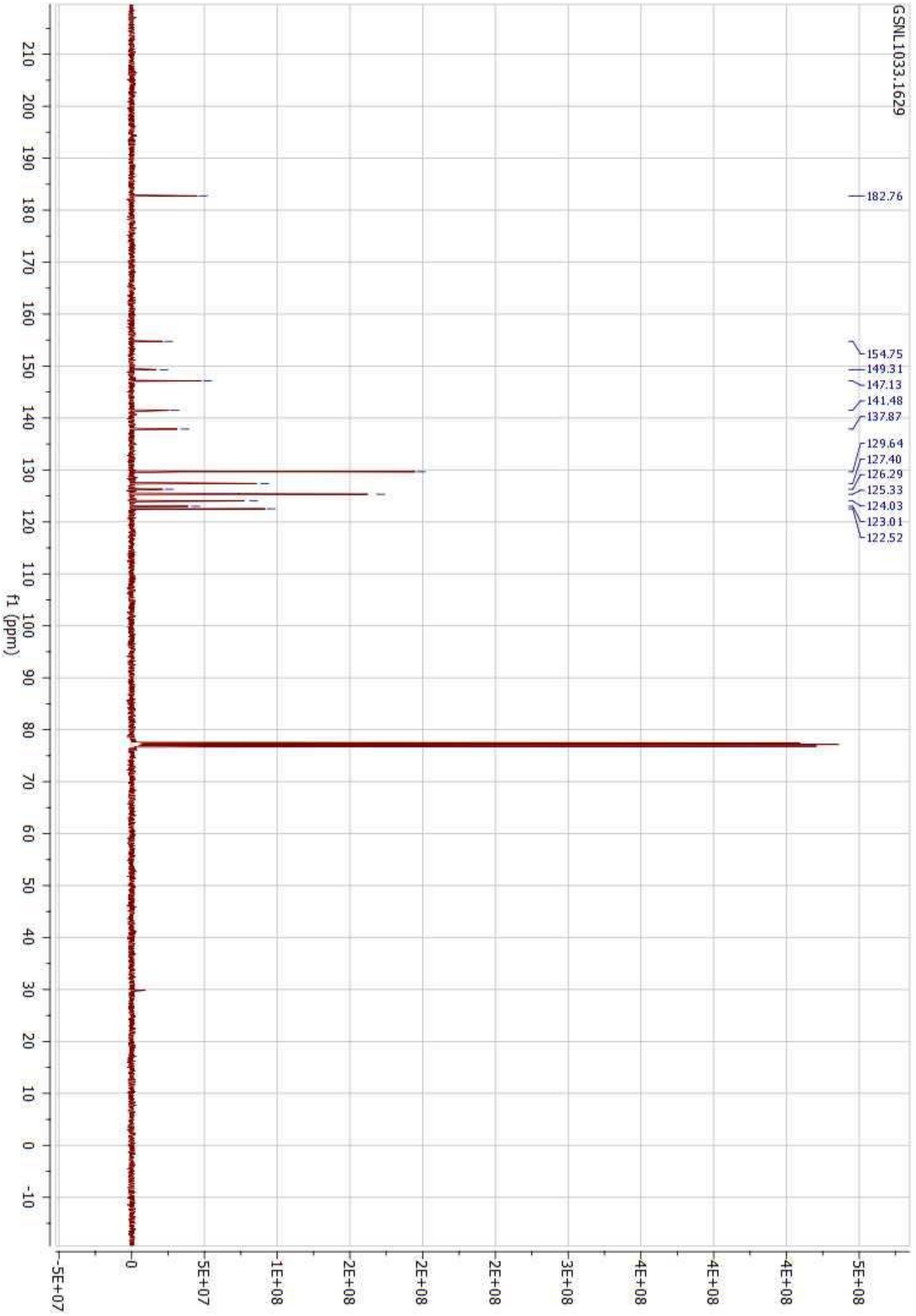

Figure S7: ^{1}H–NMR of compound 4 ($CDCl_3$, 400 MHz, 298 K)

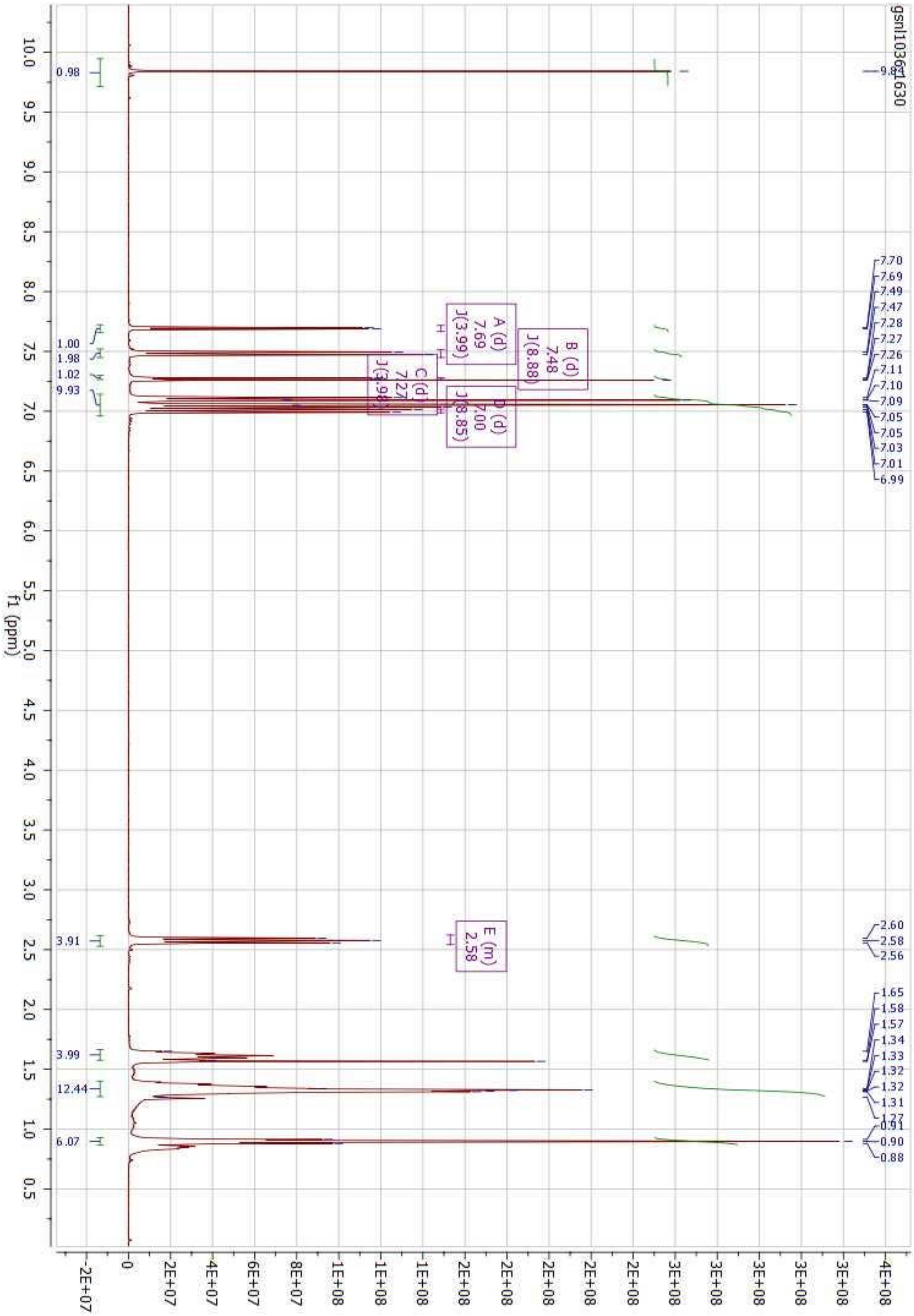

Figure S8: ^{13}C–NMR of compound 4 ($CDCl_3$, 100 MHz, 298 K)

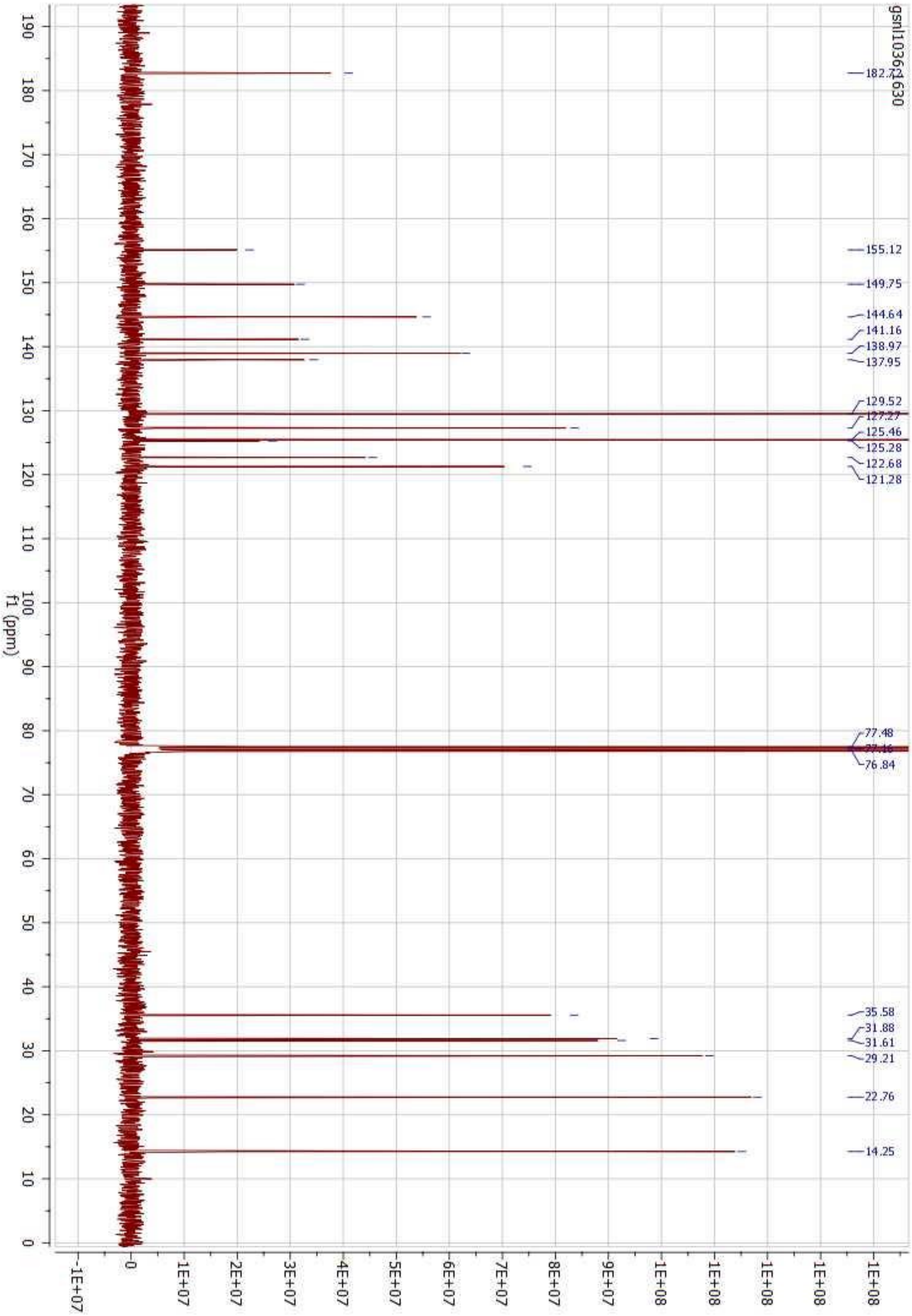

Figure S9: ^1H–NMR of compound 5 ($CDCl_3$, 400 MHz, 298 K)

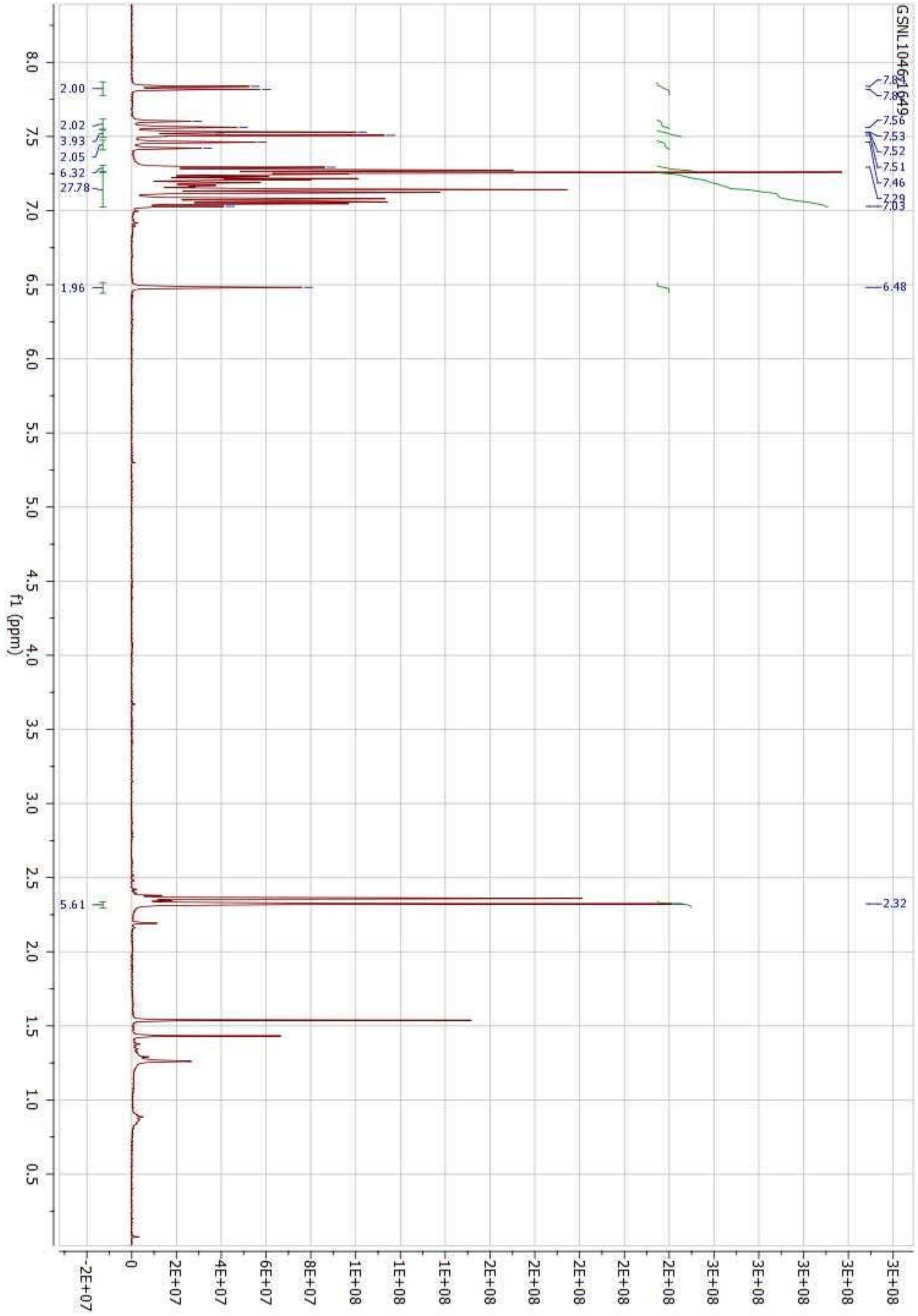

Figure S10: ^{13}C–NMR of compound 5 ($CDCl_3$, 100 MHz, 298 K)

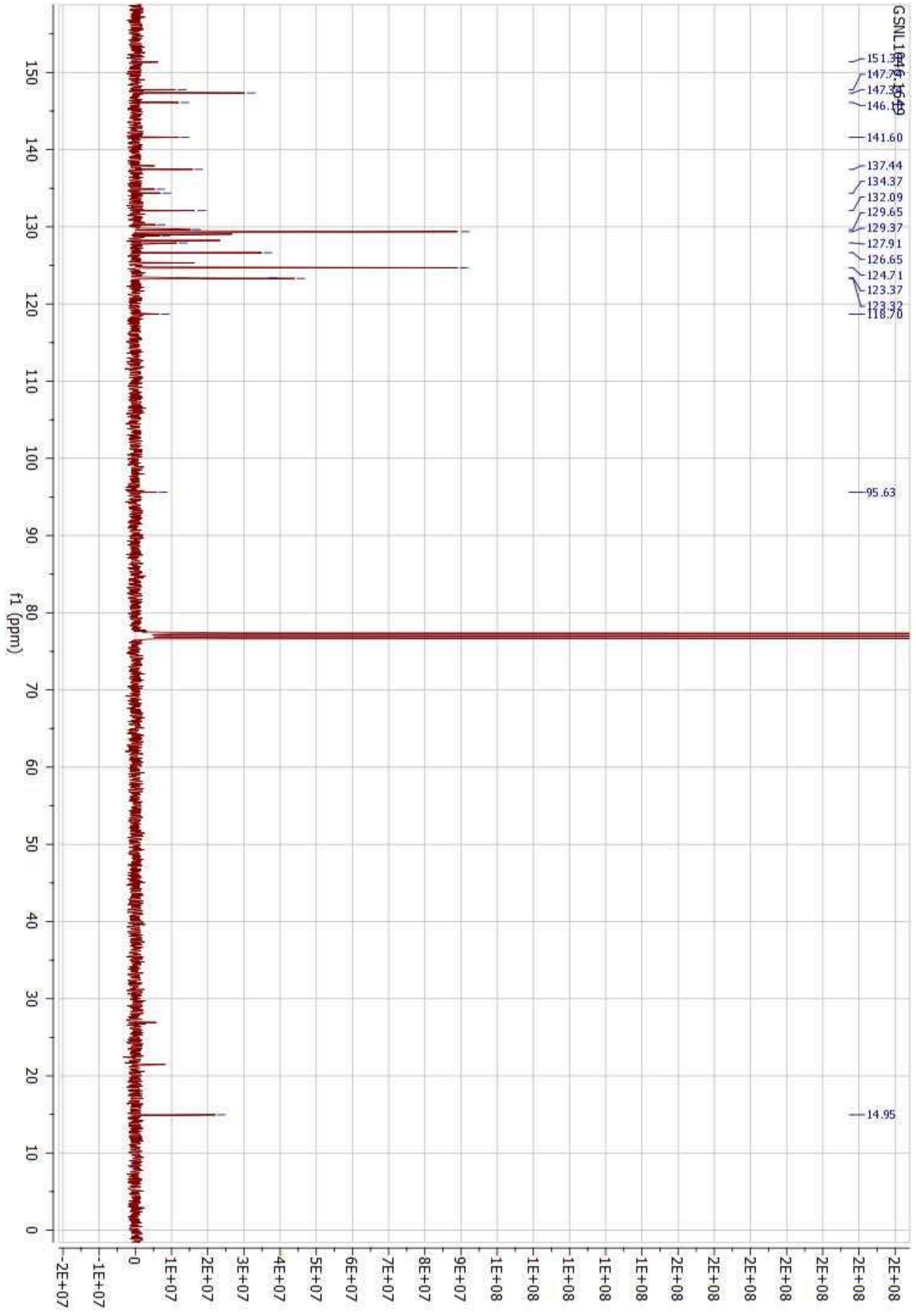

Figure S11: ^{1}H–NMR of compound 6 ($CDCl_3$, 400 MHz, 298 K)

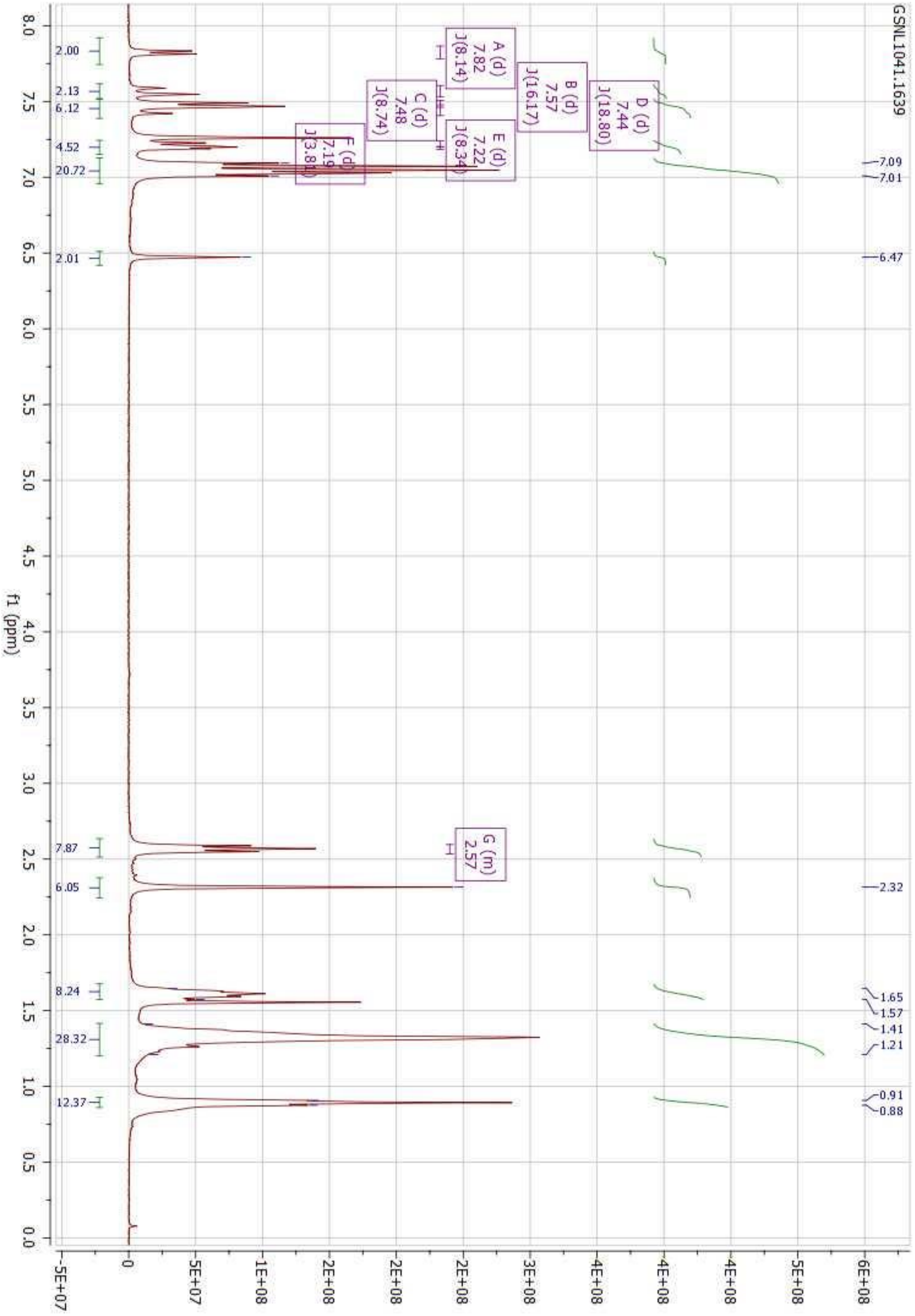

Figure S12: ^{13}C–NMR of compound 6 ($CDCl_3$, 100 MHz, 298 K)

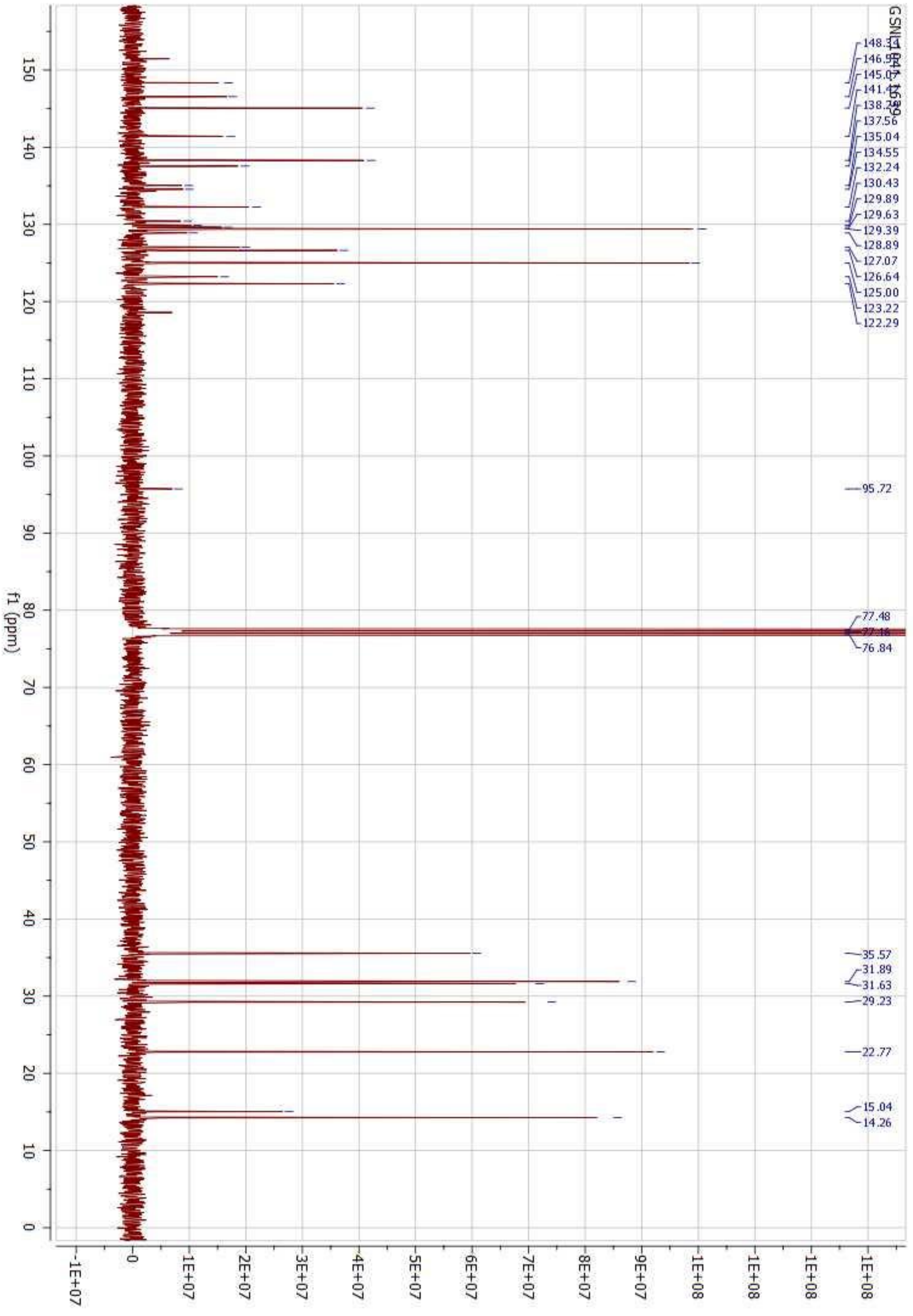

Figure S13: ^{1}H–NMR of compound 7 ($CDCl_3$, 400 MHz, 298 K)

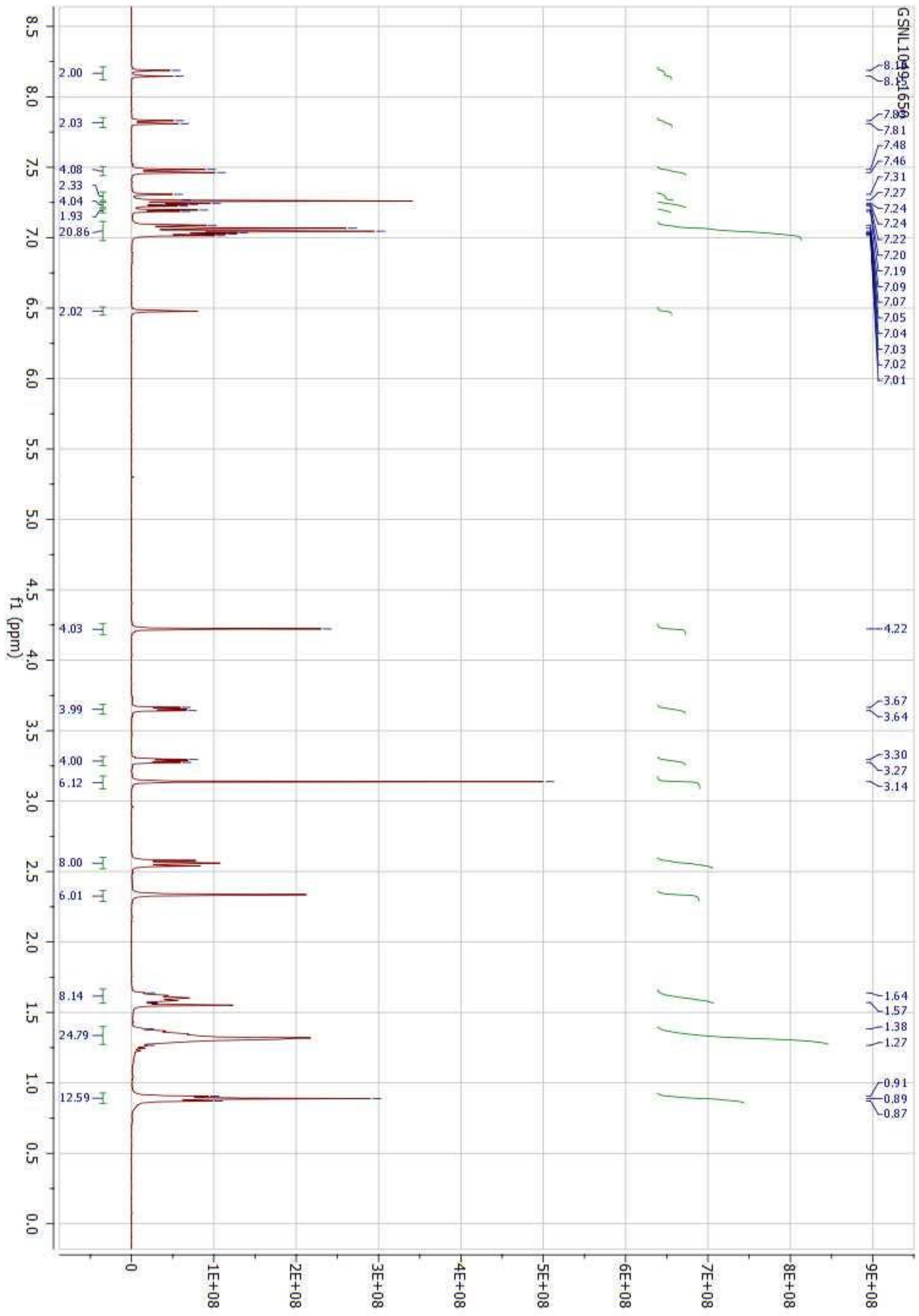

Figure S14: ^{13}C–NMR of compound 7 ($CDCl_3$, 100 MHz, 298 K)

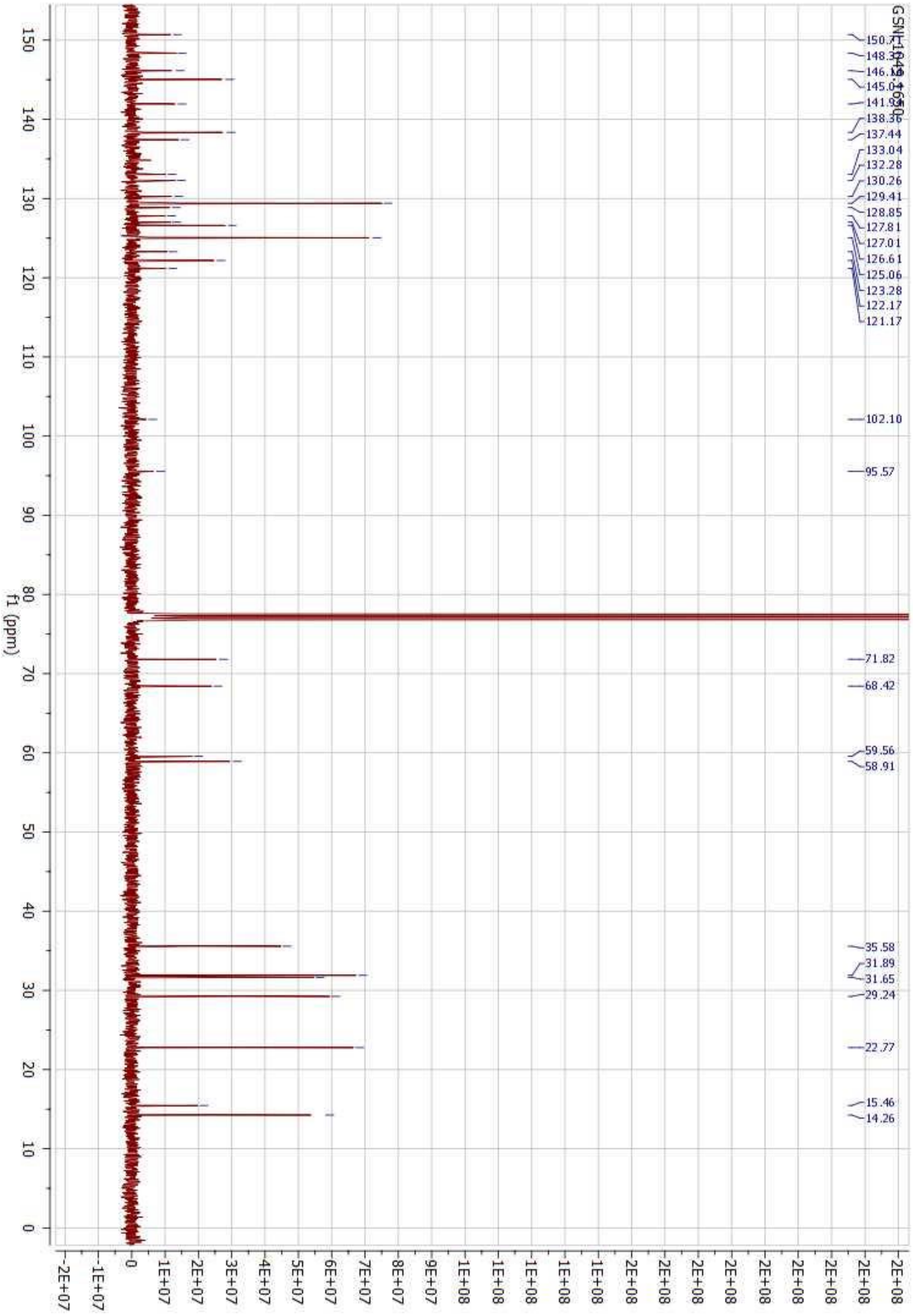

Figure S15: ^{1}H–NMR of compound 8 (CD_2Cl_2, 400 MHz, 298 K)

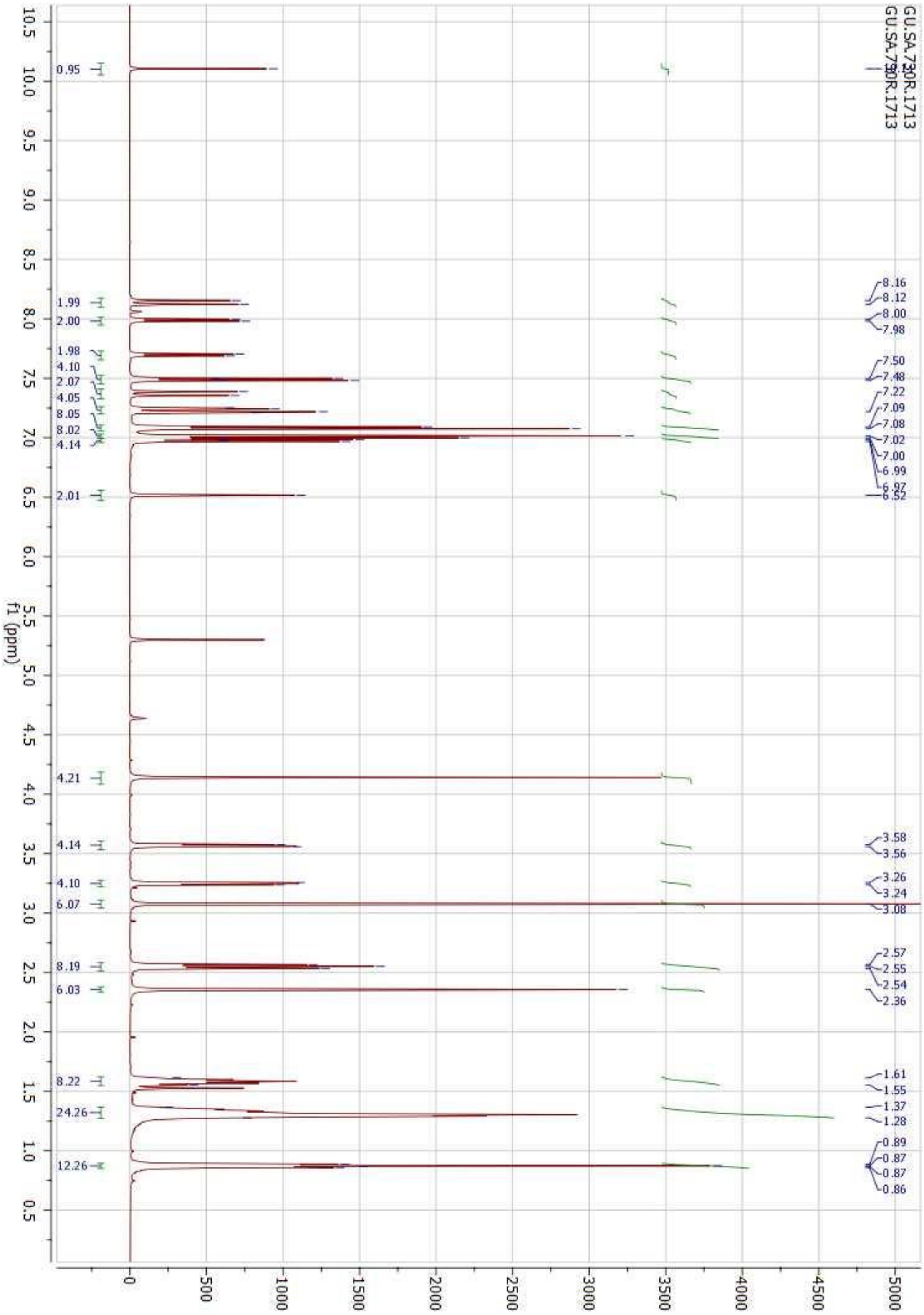

Figure S16: ^{13}C–NMR of compound 8 ($CDCl_3$, 100 MHz, 298 K)

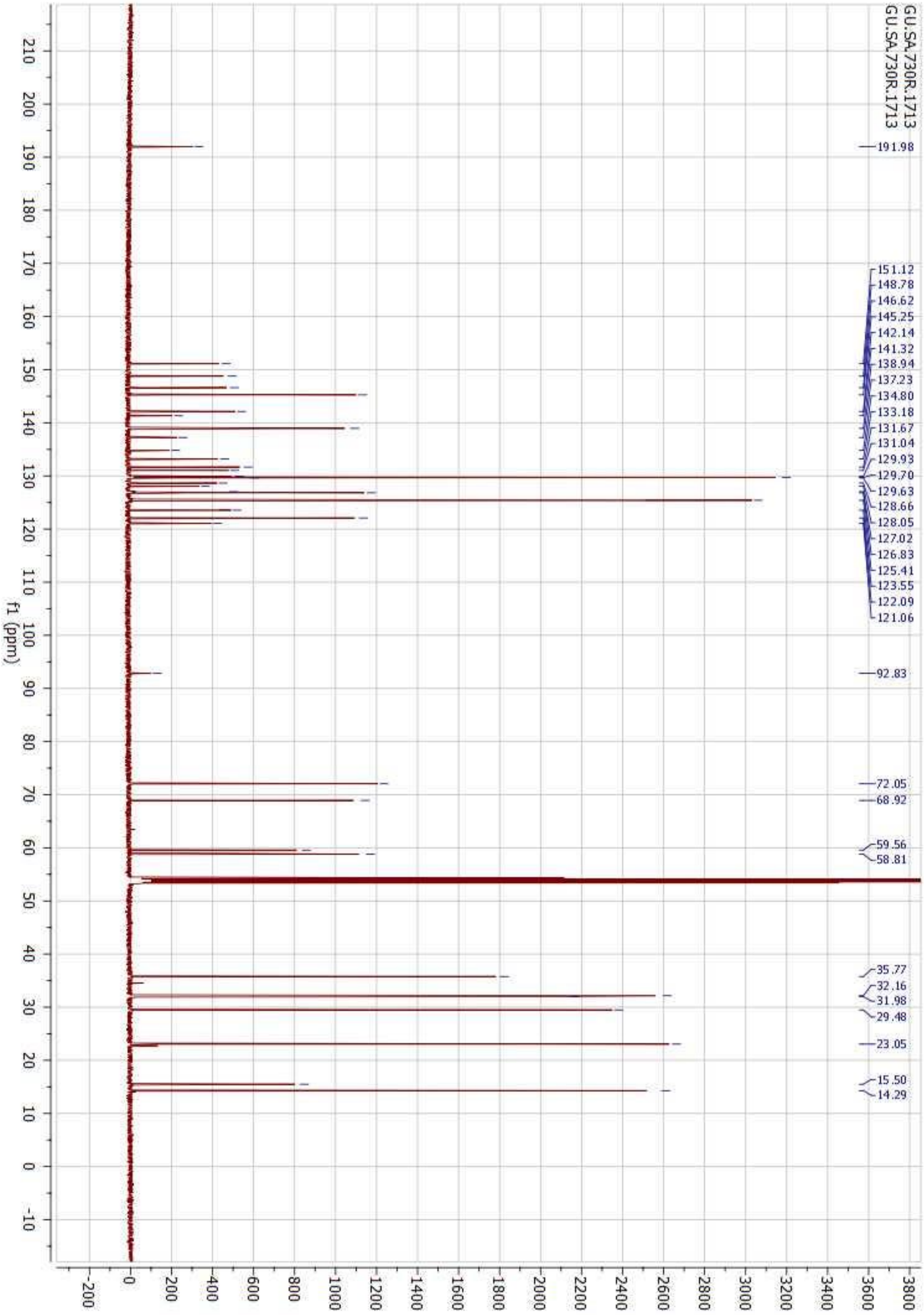

Figure S17: ¹H–NMR of compound BOD-TTPA-alk ($CDCl_3$, 400 MHz, 298 K)

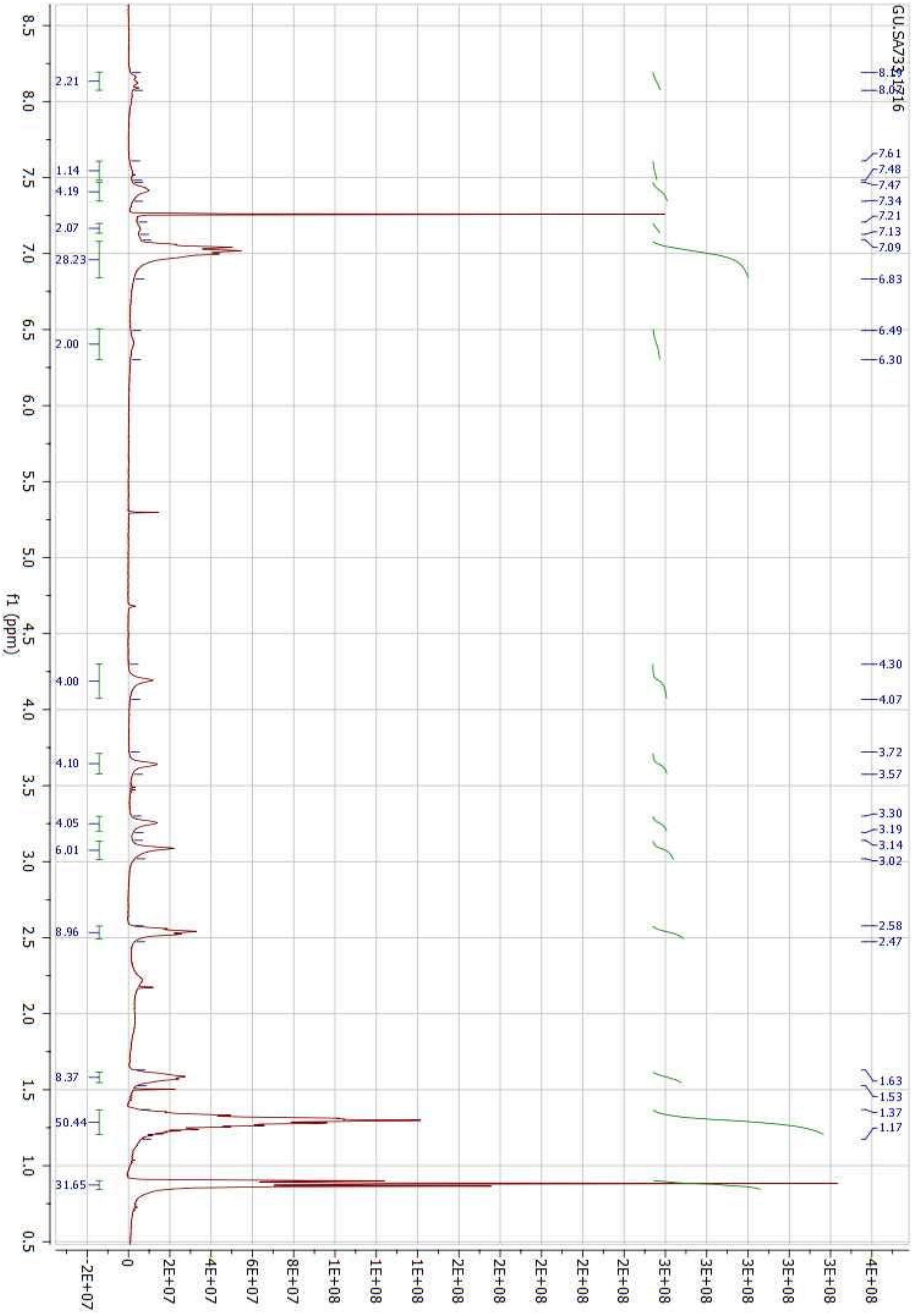

Figure S18: ^{1}H–NMR of compound 9 (CD_2Cl_2, 400 MHz, 298 K)

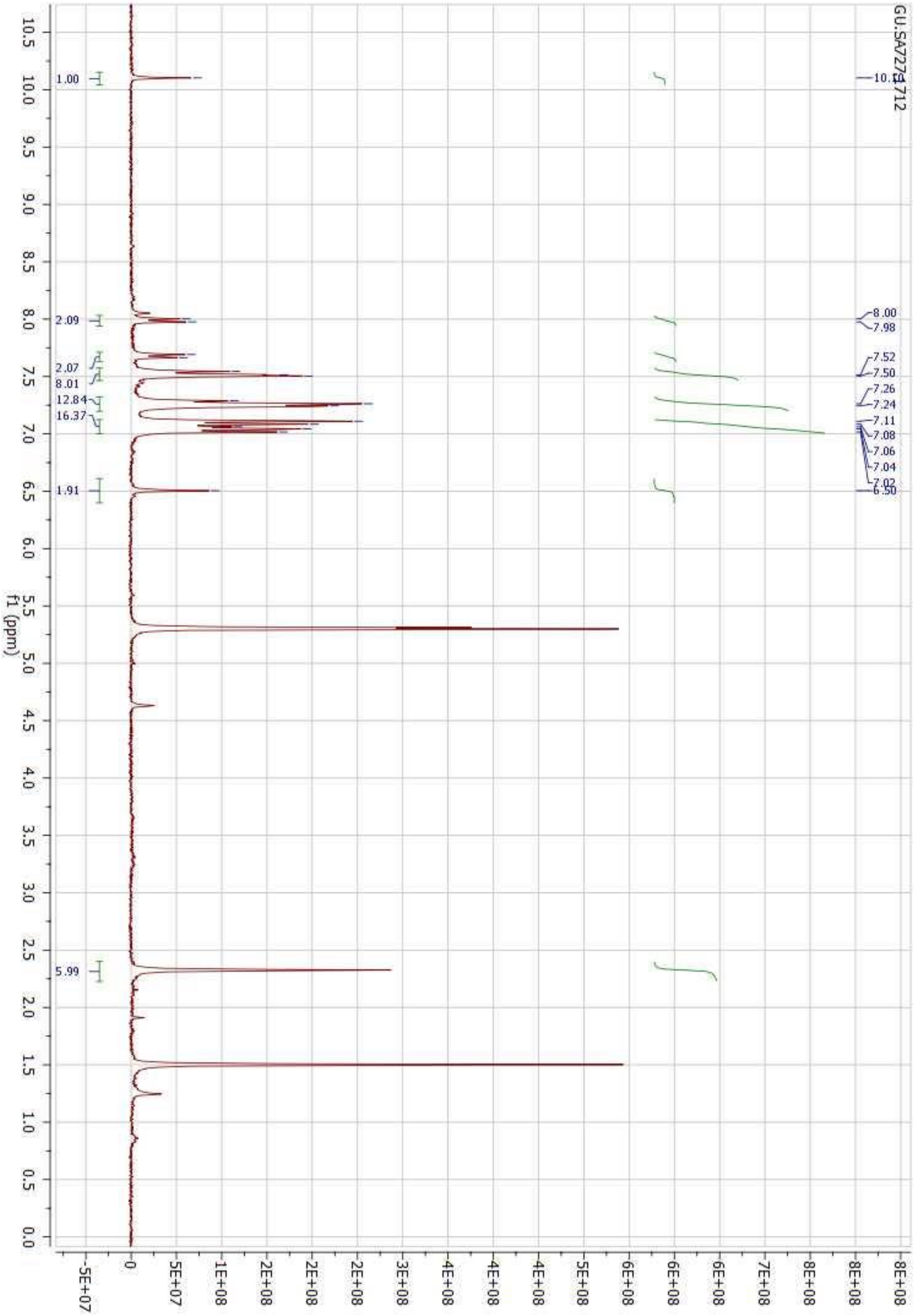

Figure S19: ^{13}C–NMR of compound 9 ($CDCl_3$, 100 MHz, 298 K)

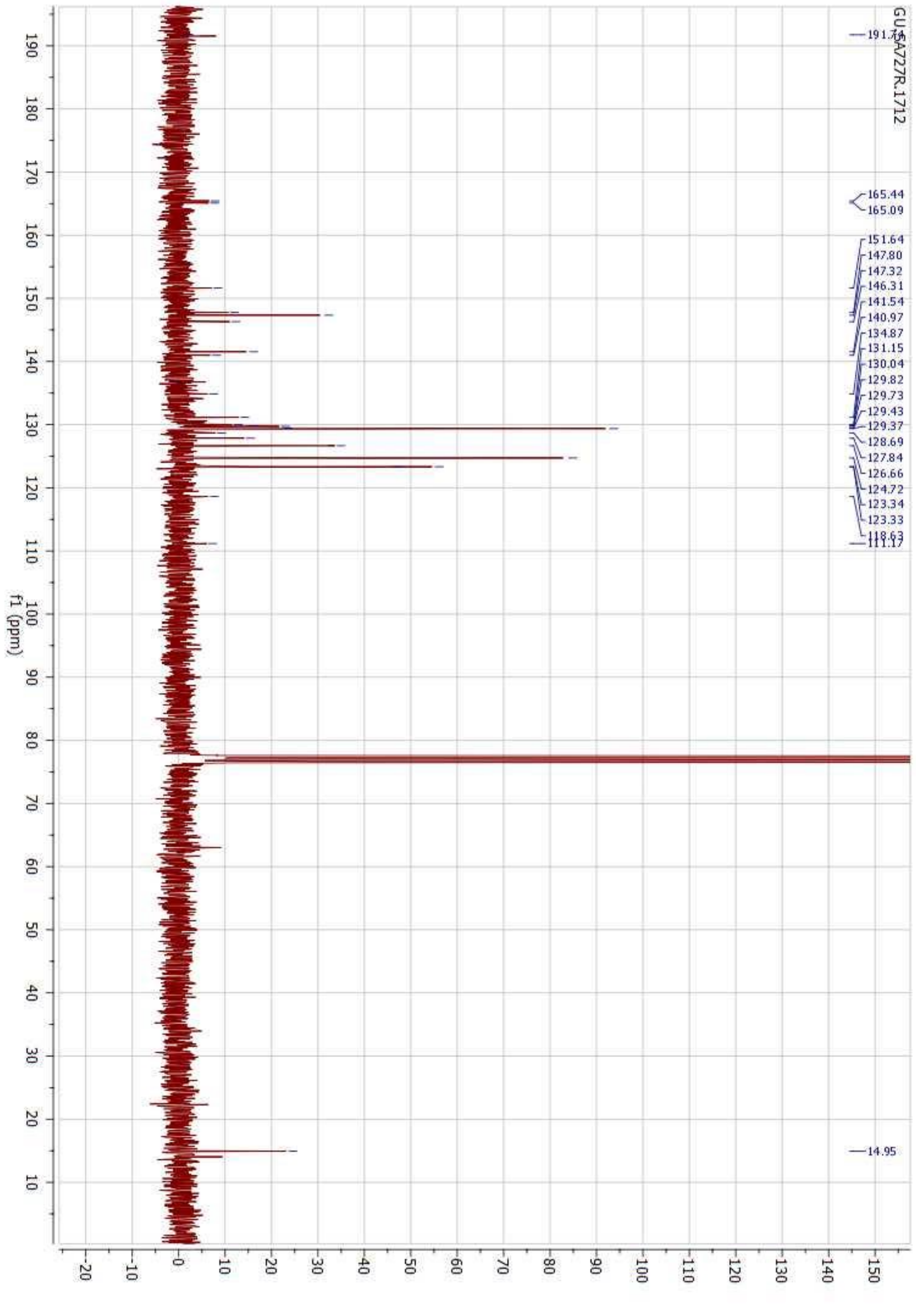

Figure S20: **^{1}H–NMR of compound BOD-TTPA ($CDCl_3$, 400 MHz, 298 K)**

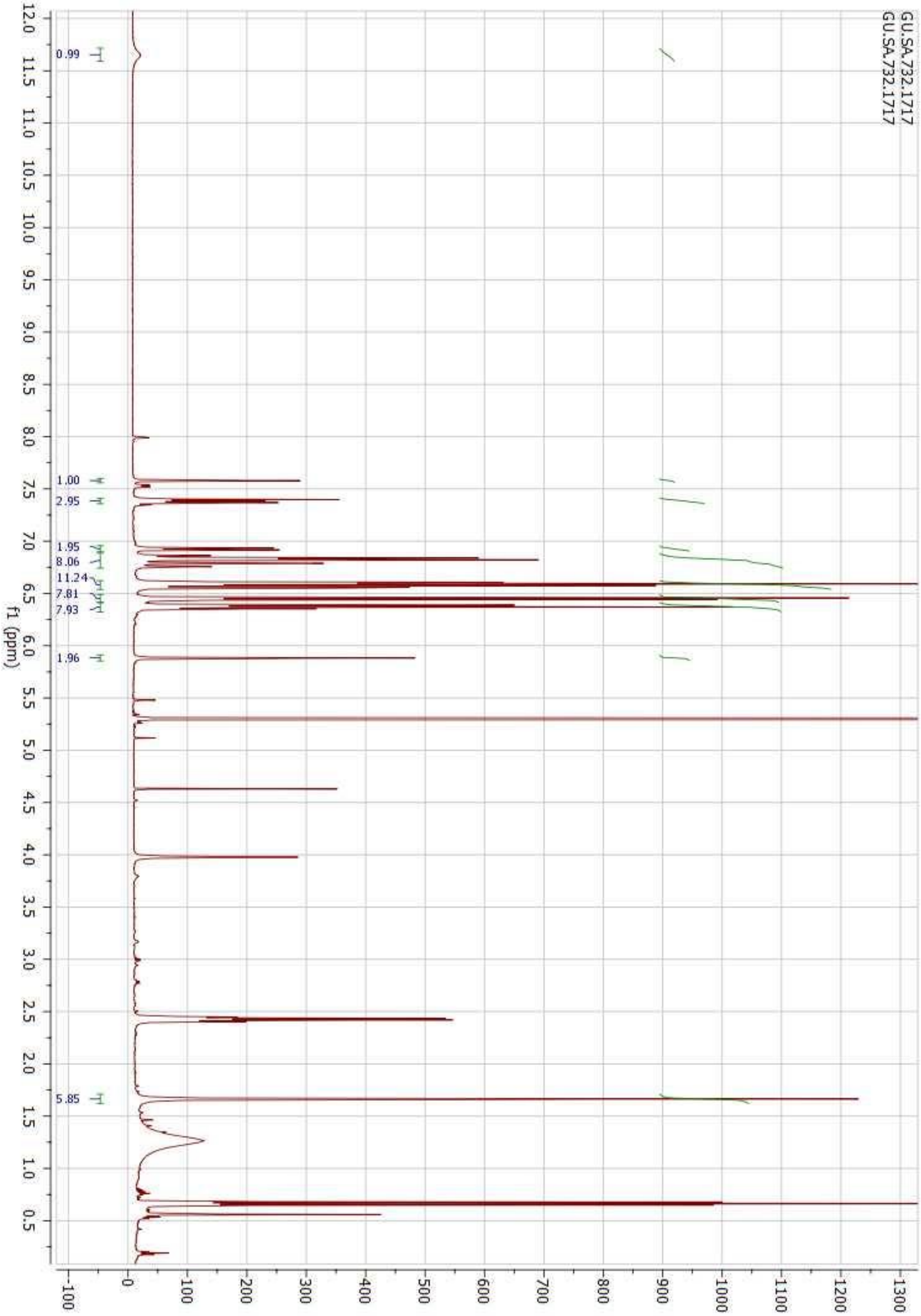

Figure S21: ^{13}C–NMR of BOD-TTPA ($CDCl_3$, 100 MHz, 298 K)

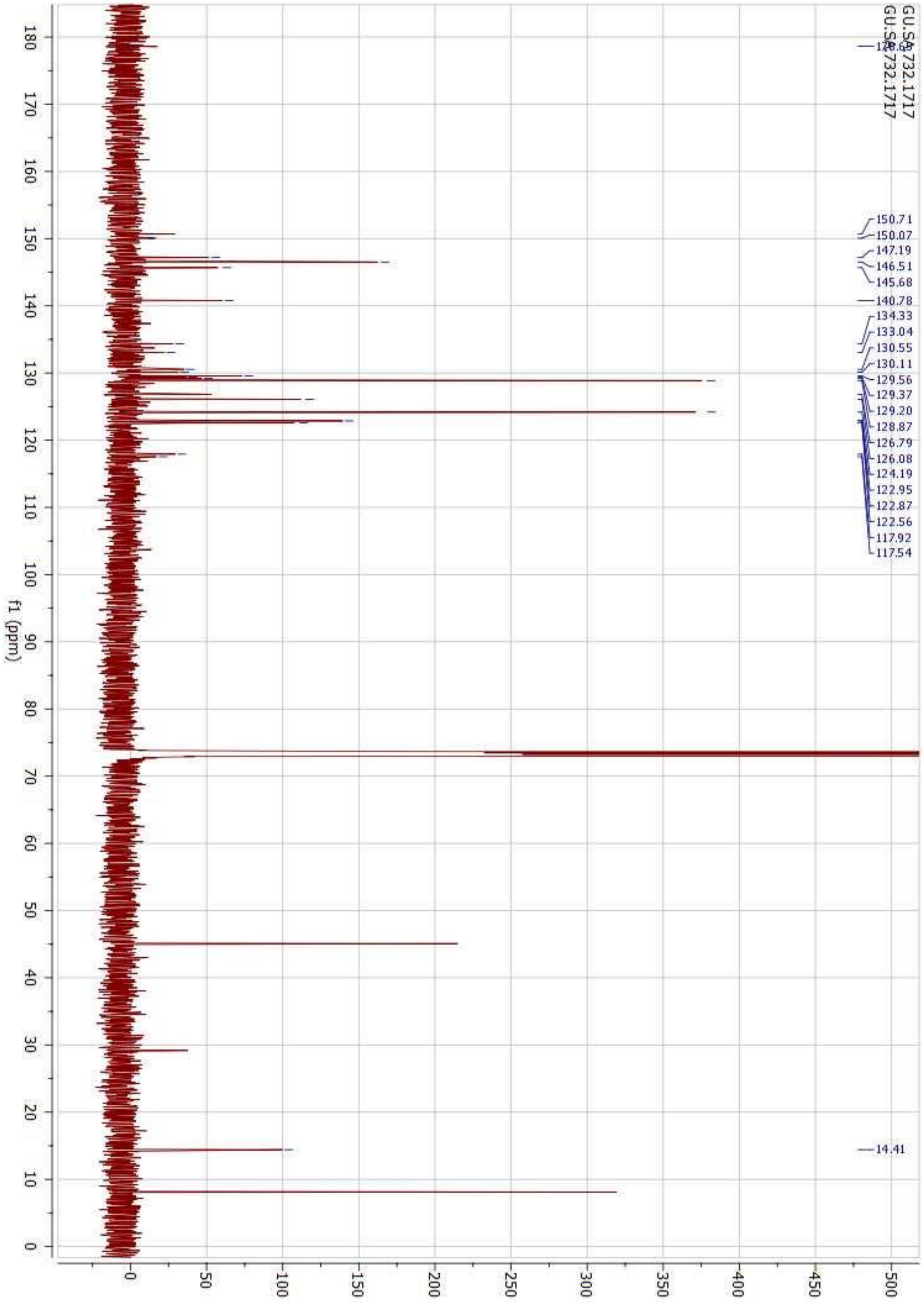

Figure S22: HRMS spectra of compound BOD-TTPA

Service de spectrometrie de masse - Institut de Chimie - Strasbourg - UMR 7177 CNRS / UDS

Analysis Info

Analysis Name	F00675SK.d	Acquisition Date	6/12/2017 2:49:20 PM
Method	Tune_pos_Mid.m	Operator	BDAL@DE
Sample Name	AS732	Instrument	micrOTOF II

Acquisition Parameter

Source Type	ESI	Capillary	4500 V	Nebulizer	0.3 Bar	Set Hexapole RF	330.0 Vpp
Ion Polarity	Positive	Dry Heater	200 °C	Dry Gas	3.0 l/min	Set Capillary Exit	150.0 V

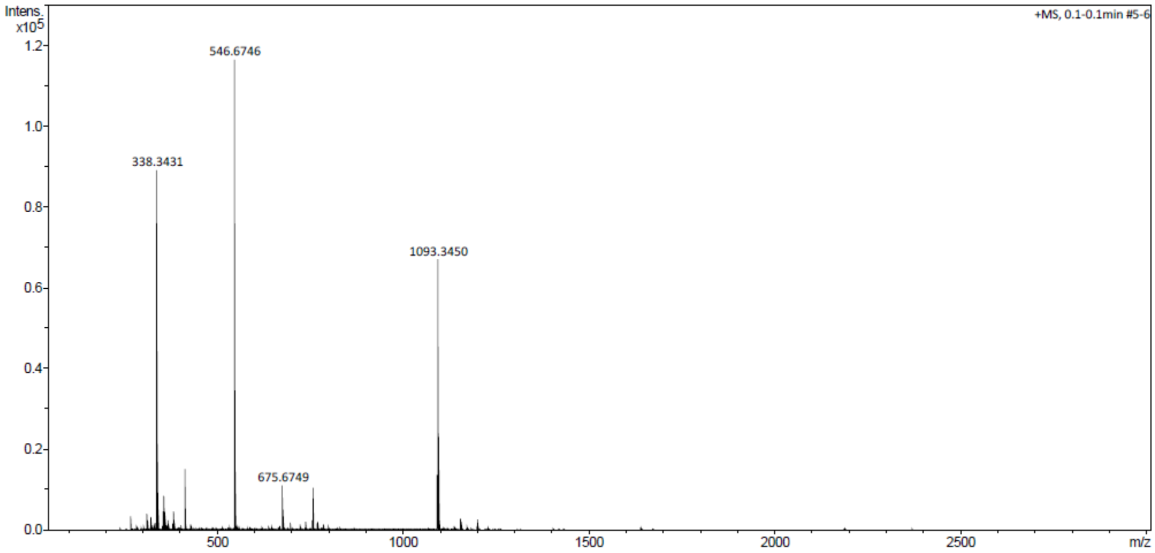

Mass Spectrum HR Report

Analysis Info

Analysis Name	D:\Data\SMasse\2017\Juin 2017\F00675SK.d	Acquisition Date	6/12/2017 2:49:20 PM	
Method	Tune_pos_Mid.m	Operator	BDAL@DE	
Sample Name	AS732	Instrument	micrOTOF II	8213750.1045 1
Comment				

Acquisition Parameter

Source Type	ESI	Ion Polarity	Positive	Set Corrector Fill	59.0 V
n/a	n/a	n/a	n/a	n/a	n/a
Scan Begin	50 m/z	n/a	n/a	Set Reflector	1800.0 V
Scan End	3000 m/z	n/a	n/a	Set Flight Tube	8600.0 V
		n/a	n/a	Set Detector TOF	1953.3 V

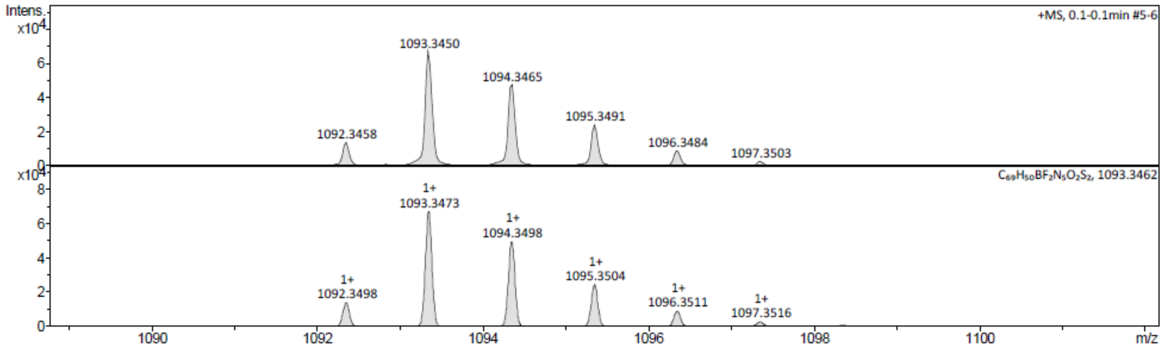

Meas. m/z	#	Ion Formula	m/z	err [ppm]	Mean err [ppm]	rdb	N-Rule	e⁻ Conf	mSigma	Std I	Std Mean m/z	Std I VarNorm	Std m/z Diff	Std Comb Dev
546.674644	1	C69H50BF2N5O2S2	546.672804	-2.4	-3.1	47.0	ok	even	5.8	5.0	n.a.	n.a.	n.a.	n.a.
	2	C69H46BF2N5O2S2	544.657154	-3703.1	-2036.5	49.0	ok	even	603.1	339.2	n.a.	n.a.	n.a.	n.a.
	3	C69H48BF2N5O2S2	545.664979	-13.8	505.6	48.0	ok	even	603.9	620.1	n.a.	n.a.	n.a.	n.a.
1093.344958	1	C69H49BF2N5O2S2	1092.338331	-2.7	-3.8	47.5	ok	even	391.0	391.9	n.a.	n.a.	n.a.	n.a.
	2	C69H51BF2N5O2S2	1094.353981	923.0	1995.6	46.5	ok	even	602.9	339.0	n.a.	n.a.	n.a.	n.a.
	3	C69H47BF2N5O2S2	1090.322681	-2770.9	-1224.0	48.5	ok	even	603.1	339.1	n.a.	n.a.	n.a.	n.a.
	1	C69H50BF2N5O2S2	1093.346156	2.1	2.3	47.0	ok	odd	14.8	11.6	n.a.	n.a.	n.a.	n.a.

Figure S23: HRMS spectra of compound BOD-TTPA-alk

Analysis Info

Analysis Name	F00680SK.d	Acquisition Date	6/13/2017 10:39:32 AM
Method	Tune_pos_Mid.m	Operator	BDAL@DE
Sample Name	AS733	Instrument	micrOTOF II

Acquisition Parameter

Source Type	ESI	Capillary	4500 V	Nebulizer	0.3 Bar	Set Hexapole RF	330.0 Vpp
Ion Polarity	Positive	Dry Heater	200 °C	Dry Gas	3.0 l/min	Set Capillary Exit	150.0 V

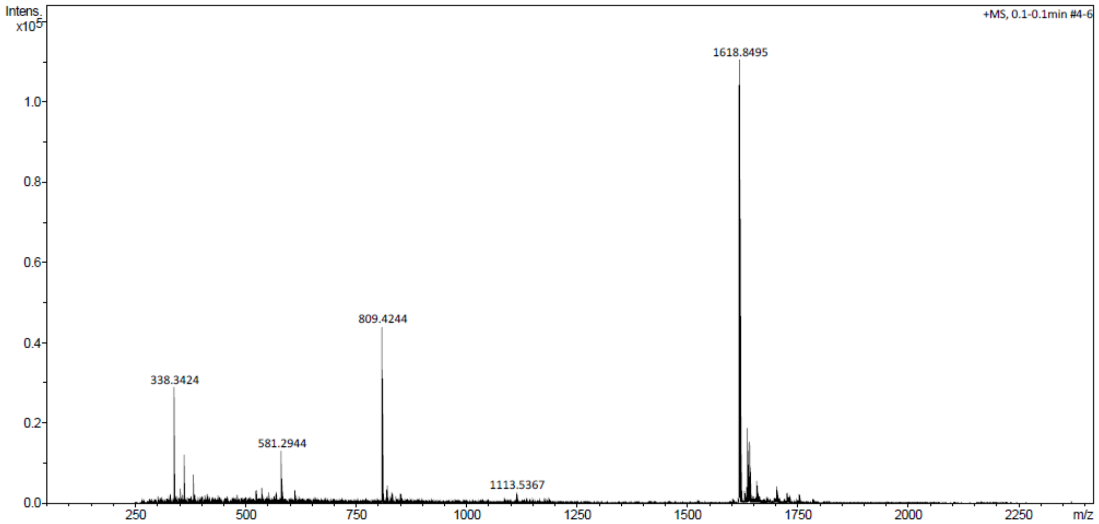

Mass Spectrum HR Report

Analysis Info

Analysis Name	D:\Data\SMasse\2017\Juin 2017\F00680SK.d	Acquisition Date	6/13/2017 10:39:32 AM
Method	Tune_pos_Mid.m	Operator	BDAL@DE
Sample Name	AS733	Instrument	micrOTOF II 8213750.10451
Comment			

Acquisition Parameter

Source Type	ESI	Ion Polarity	Positive	Set Corrector Fill	59.0 V
n/a	n/a	n/a	n/a	n/a	n/a
Scan Begin	50 m/z	n/a	n/a	Set Reflector	1800.0 V
Scan End	3000 m/z	n/a	n/a	Set Flight Tube	8600.0 V
		n/a	n/a	Set Detector TOF	1953.3 V

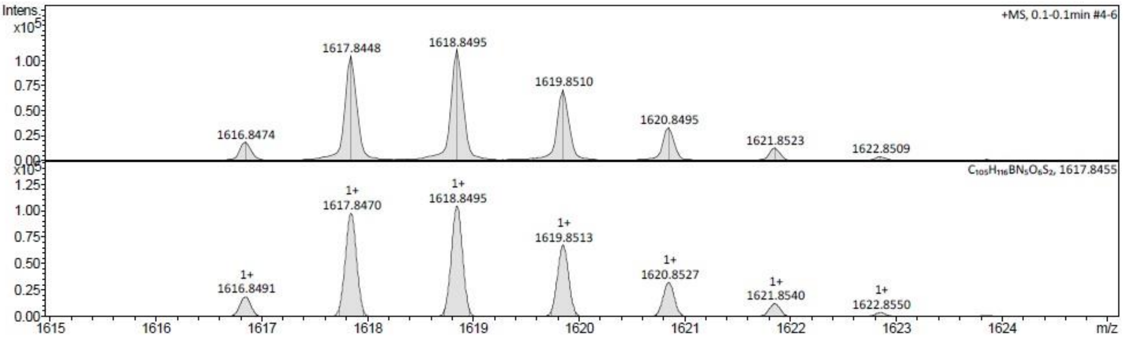

Meas. m/z	#	Ion Formula	m/z	err [ppm]	Mean err [ppm]	rdb	N-Rule	e⁻ Conf	mSigma	Std I	Std Mean m/z	Std I VarNorm	Std m/z Diff	Std Comb Dev
1615.824444	1	C105H114BN5O6S2	1615.829810	1.$	-621.3	52.0	ok	odd	578.5	293.1	n.a.	n.a.	n.a.	n.a.
	1	C105H114BN5O6S2	1615.829810	1.$	-621.3	52.0	ok	odd	578.5	293.1	n.a.	n.a.	n.a.	n.a.
1616.847375	1	C105H113BN5O6S2	1614.821985	1.$	-1183.1	52.5	ok	even	578.5	293.1	n.a.	n.a.	n.a.	n.a.
	2	C105H111BN5O6S2	1612.806335	1.$	-2308.7	53.5	ok	even	578.5	293.2	n.a.	n.a.	n.a.	n.a.
	3	C105H109BN5O6S2	1610.790685	1.$	-3437.1	54.5	ok	even	578.5	293.2	n.a.	n.a.	n.a.	n.a.
1617.844793	1	C105H115BN5O6S2	1616.837635	-1.9	-3.0	51.5	ok	even	358.0	296.2	n.a.	n.a.	n.a.	n.a.
	2	C105H117BN5O6S2	1618.853285	1.$	1059.8	50.5	ok	even	578.4	293.1	n.a.	n.a.	n.a.	n.a.

Cyclic voltammetry measurements

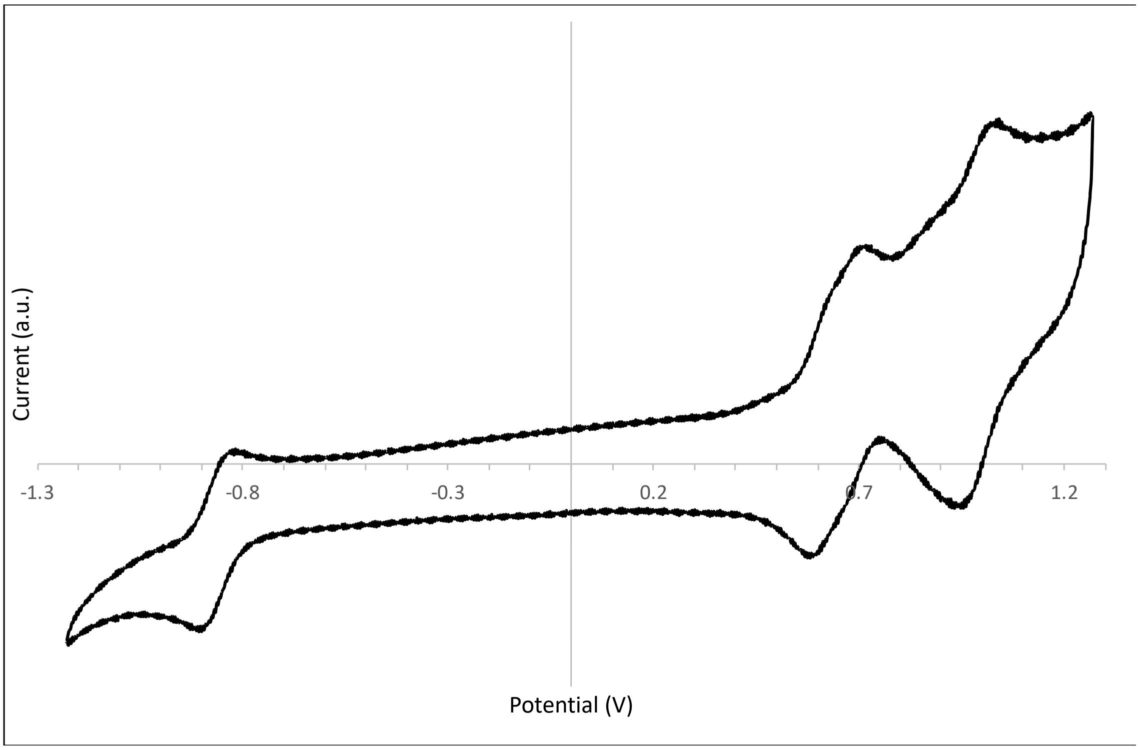

Figure S24: CV measurement of the BOD-TTPA-alk. Scan speed: 100 mV/s, scan direction: from 0 V to 1.2 V to −1.3 V.

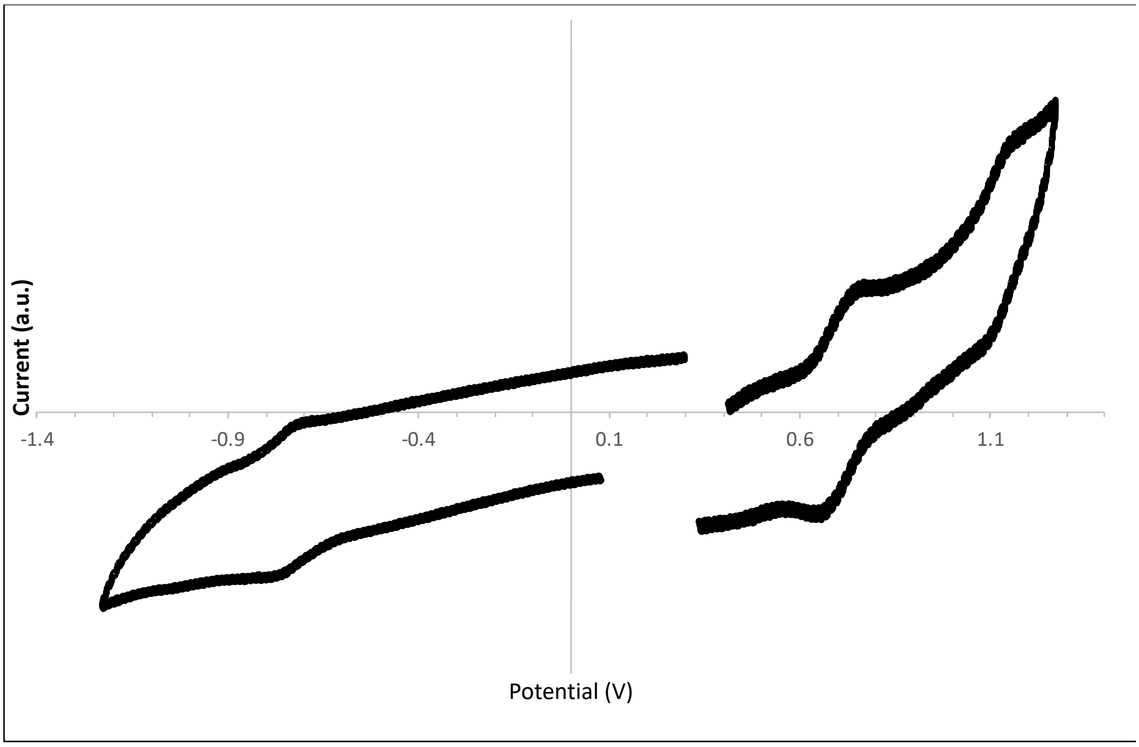

Figure S25: CV measurement of the BOD-TTPA. Scan speed: 100 mV/s. scan direction: from 0 V to 1.2 V to −1.2 V.

Devices electrical parameters

Table S1: Devices electrical parameters as a function of tBP content in the electrolyte (0.5M of BMII, 0.03M of I2 0.5M of LiI, 0.1M of guanidinium thiocyanate in a mixture of acetonitrile and 3-methoxyproprionitrile (85:15, v/v))

Dye	[^{t}BP] (mol/L)	J_{sc} (mA·cm^{-2})	V_{oc} (V)	FF	PCE (%)
**BOD-TTPA-alk**	0	4.70	0.37	0.65	1.12
	0.1	1.48	0.38	0.61	0.27
	0.5	0.41	0.42	0.61	0.10
**BOD-TTPA**	0	7.33	0.33	0.51	1.22
	0.1	2.70	0.39	0.66	0.69
	0.5	0.88	0.46	0.67	0.27

References

[1]. A. P. Kulkarni; C. J. Tonzola; A. Babel; S. A. Jenekhe; *Chem. Mater.***2004**, 16, 23, 4556-4573

[2]. T. Marinado, D. P. Hagberg, M. Hedlund, T. Edvinsson, E. M. J. Johansson, G. Boschloo, H. Rensmo, T. Brinck, L. Sun and A. Hagfeldt; *PhysChemChemPhys*, **2009**, 133-141.

[3]. A. Poirel; A. De Nicola; P. Retailleau; R. Ziessel; *The Journal of Organic Chemistry,* **2012**, 77, 17, 7512-7525.
